# PAK1 Promotes Gluconeogenesis Via the ACSS2/KAT2A/PCK1 Complex to Attenuate Steatotic Liver Ischemia-Reperfusion Injury

**DOI:** 10.7150/ijbs.132770

**Published:** 2026-09-11

**Authors:** Fengqiang Gao, Ye Tao, Zijian Lou, Jinjian Li, Libin Dong, Yuhang Huang, Xiao Tang, Hanzhi Xu, Zhoucheng Wang, Shusen Zheng, Lixing Zhan, Xinyu Yang, Shengjun Xu, Kai Wang, Xiao Xu

**Affiliations:** 1 Hepatobiliary Center and Transplantation Center, The First Affiliated Hospital with Nanjing Medical University, Nanjing 210029, China.; 2 Institute of Translational Medicine, Zhejiang University School of Medicine, Hangzhou 310000, China.; 3 Department of General Surgery, Sir Run Run Shaw Hospital, School of Medicine, Zhejiang University, Hangzhou 310016, China.; 4 Department of Hepatobiliary and Pancreatic Surgery, Shulan (Hangzhou) Hospital, Hangzhou 310022, China.; 5 Key Laboratory of Nutrition, Metabolism, and Food Safety, Institute of Nutrition and Health, Shanghai Institutes for Biological Sciences, University of the Chinese Academy of Sciences, Shanghai 200031, China.; 6 Department of Hepatobiliary and Pancreatic Surgery, Affiliated Hangzhou First People's Hospital, School of Medicine, Westlake University, Hangzhou 310006, China.; 7 General Surgery, Cancer Center, Department of Hepatobiliary & Pancreatic Surgery and Minimally Invasive Surgery, Zhejiang Provincial People's Hospital (Affiliated People's Hospital), Hangzhou Medical College, Hangzhou 310024, China.

**Keywords:** p21-activated kinase 1, steatotic liver, gluconeogenesis, lactylation

## Abstract

Ischemia-reperfusion (IR) injury is particularly severe in steatotic livers, substantially increasing the risk of graft failure. The growing use of steatotic grafts amid donor shortages has therefore heightened the clinical importance of this problem. The conserved serine/threonine kinase p21-activated kinase 1 (PAK1) regulates cell migration, growth, and redox homeostasis, but its role in steatotic liver IR injury remains unclear. To address this question, we examined the association between PAK1 expression in human steatotic liver grafts and clinical outcomes in 74 liver transplantation (LT) recipients. Liver function, inflammation, and apoptosis were assessed using liver enzyme assays, F4/80 and Ly-6G immunostaining, and TUNEL staining, respectively. Co-immunoprecipitation and RNA sequencing were performed to elucidate the underlying molecular mechanisms. High PAK1 levels in steatotic liver grafts were associated with improved graft function and recipient survival after LT. Consistent with these clinical findings, PAK1 knockout exacerbated IR-induced hepatic inflammation and hepatocyte apoptosis, whereas PAK1 overexpression conferred protection; these findings were corroborated *in vitro*. Mechanistically, PAK1 interacted directly with ACSS2 and mediated ACSS2 phosphorylation at S267. Phosphorylated ACSS2 subsequently recruited KAT2A to form a multiprotein complex that locally catalyzed PCK1 K547 lactylation, thereby promoting gluconeogenesis. This process suppressed reactive oxygen species (ROS) production and enhanced energy supply. Moreover, fingolimod effectively protected steatotic livers against IR injury in the experimental model. Together, these findings highlight PAK1 as an important regulator of gluconeogenesis through the ACSS2-KAT2A-PCK1 complex and provide new insights into therapeutic strategies for protecting steatotic liver grafts against IR injury.

## Introduction

Hepatic ischemia-reperfusion (IR) injury is a major cause of liver dysfunction following temporary interruption and restoration of hepatic blood flow. IR injury is a major clinical challenge during liver transplantation (LT) and an important determinant of graft function and recipient outcomes^1^. However, due to the shortage of donor organs, the clinical use of expanded-criteria grafts, particularly steatotic livers, has increased^2^, ^3^. Steatotic grafts exhibit heightened vulnerability to IR injury, characterized by excessive reactive oxygen species (ROS) generation and amplified pro-inflammatory cytokine release, which exacerbate hepatocellular apoptosis and impair graft function^4^, ^5^.

p21-activated kinases (PAKs) are a six-member, CDC42/Rac-responsive serine/threonine kinase family that regulates proliferation, survival, and cytoskeletal dynamics via PI3K-AKT, Wnt/β-catenin, and related signaling^6^, ^7^. PAK4 has been reported to be upregulated during hepatic IR injury, and its inhibition mitigates hepatocellular inflammation and necrosis by enhancing the expression of nuclear factor erythroid 2-related factor 2 (Nrf2) 8. However, although PAK1 expression has been observed to increase in both steatotic and non-steatotic grafts post-transplant, its role and mechanisms in hepatic IR injury, especially in fatty liver remain undefined.

L-lactate is recognized as an important metabolic intermediate. The identification of L-lactate driven protein lactylation as a post-translational modification has uncovered a signal axis that assigns lactate a direct role in cell communication^9^, ^10^. This modification regulates diverse cellular programs and operates across multiple physiological and pathological contexts^11^. Thus, protein lactylation may serve as a key integrator of metabolic and signal pathways and as a therapeutic target across a range of diseases^12^. Activation of the methyltransferase 3/N6-methyladenosine-phosphoenolpyruvate carboxykinase 1 (PCK1) axis has been reported to be essential for protection against hepatic IR injury by promoting the gluconeogenesis, thereby reducing hepatocellular energy loss and enhancing glycogen storage for utilization^13^. Meanwhile, Lys100 lactylation of PCK2 promotes ferroptosis during IR injury by blocking Parkin-mediated polyubiquitination of 3-oxoacyl-ACP synthase (OXSM) resulting in metabolic reprogramming^14^. However, it remains unclear whether PCK1 is subject to lactylation that influences hepatic IR injury, and whether this modification is regulated by PAK1.

In this study, we found increased PAK1 expression in steatotic liver grafts and it was associated with improved liver function. PAK1 knockout was observed to exacerbate IR-induced hepatic injury, while PAK1 overexpression produced protective effects. Our findings indicate that PAK1 interacted directly with ACSS2 to mediate the S267 phosphorylation. The phosphorylated ACSS2 then recruited KAT2A to form the multiprotein complex that catalyzed PCK1 lactylation locally, therefore promoting gluconeogenesis to suppress ROS production and enhanced energy supply. In addition, Fingolimod was proved to protect against steatotic IR injury effectively.

## Materials and Methods

### Clinical samples

To analyze the general exchange of PAK1 level between the cold ischemia and the post-reperfusion stages, this study included the steatotic livers in the Shulan (Hangzhou) Hospital. All patients provided their written informed consent. The Ethics Committee of Shulan (Hangzhou) Hospital gave the approval for this research, which complied with the 1975 Helsinki Declaration's ethical principles (No. KY2023029). Baseline characteristics and clinicopathological features were collected of all recipients. All liver grafts were only obtained from donation after cardiac or brain death.

### High-fat diet (HFD) induced mouse model of metabolic dysfunction-associated fatty liver disease

To induce metabolic dysfunction-associated fatty liver disease (MAFLD), all the mice were maintained on a HFD providing 60% of total caloric intake from fat (D12492, Research Diets) for 8 weeks, leading to the development of moderate hepatic steatosis. According to the manufacturer's formulation, D12492 provides 60% kcal from fat and 20% kcal from carbohydrates, with no free fructose added as a separate ingredient. The carbohydrate sources are Lodex 10 (125.0 g per 773.85 g diet) and sucrose (72.8 g per 773.85 g diet). Because sucrose accounts for approximately 7.2% of total energy, the fructose-equivalent contribution derived from sucrose is approximately 3.6% of total kcal.

### Administration of AAV8 vectors

AAV8 vectors designed for hepatocyte-specific PAK1 overexpression and silencing were generated by Vigene Biosciences Co., Ltd. (Shandong, China). To achieve selective expression of PAK1 in hepatocytes, the full-length PAK1 coding sequence was inserted into an AAV8 backbone under the control of the cytomegalovirus immediate-early (CMV) promoter. For PAK1 knockdown, an AAV8 vector encoding a triple short-hairpin RNA (shRNA) sequence based on the miR30 scaffold and driven by the CMV promoter was employed. As a negative control, mice received an AAV8-CMV construct carrying a null cassette, meaning that no functional gene was expressed. Finally, viral delivery was performed via tail vein injection, with each mouse receiving 2 × 10¹¹ vector genomes suspended in a total volume of 100 μL one month before the mouse hepatic IR.

### Mouse hepatic IR injury model

A mouse model of partial (70%) hepatic warm IR injury was established as previously described^15^. In brief, the portal triad structures supplying the cephalad liver lobes, including the hepatic artery, portal vein, and bile duct, were occluded using a microvascular clamp for 1.5 h. Reperfusion was initiated immediately after clamp removal. Mice in the sham group underwent the same surgical exposure without vascular obstruction. For therapeutic intervention, HFD-fed mice received intraperitoneal injections of specific compounds one day prior to hepatic ischemia. These included FRAX597 (5 mg/kg, i.p., S7271, Selleck), 3-MPA (35 mg/kg, i.p., HY-128923, MCE), MB-3 (15 mg/kg, i.p., HY-129039, MCE), FTY720 (15 mg/kg, i.p., HY-12005, MCE) or SEW2871 (10 mg/kg, i.p., HY-W008947, MCE). All agents were prepared in a vehicle solution consisting of DMSO, PEG300, Tween-80, and water at a ratio of 4:30:5:61. Animals were euthanized 6 h following reperfusion, and both ischemic liver lobes and serum samples were harvested for subsequent analyses.

### Liver biochemical measurement

Serum biochemical indicators, including alanine aminotransferase (ALT), aspartate aminotransferase (AST), total cholesterol (TC), and triglycerides (TG), were analyzed using the Chemray 800 automatic biochemical analyzer (Shenzhen, China). To assess the inflammatory responses, serum cytokine levels were quantified. This was achieved with enzyme-linked immunosorbent assay (ELISA) kits specific for IL-6 (Mouse Uncoated ELISA Kit, 88-7064-88), IL-1β (Mouse Uncoated ELISA Kit, 88-7013-88), and TNF-α (Mouse Uncoated ELISA Kit, 88-7324-22), all obtained from Invitrogen.

### DNA construction and transfection

Plasmids were obtained from Wuhan Miaoling Biotechnology Co., Ltd. (Miaoling Bio, Wuhan, China). Detailed information was provided in **Supplementary Table S1**. Plasmids were purified with endotoxin-free kits when applicable, and quantified spectrophotometrically (A260/280 =1.8-2.0). Working stocks were stored at -20 °C. AML12 and HEK-293T cells were seeded in six-well plates at 2 × 10^5^ cells/well. After reaching approximately 60% confluence, the cells were transfected with the specific plasmids using Polyplus transfection reagent (jetPRIME) for 48 h.

### Quantitative real-time PCR and Western Blot analysis

Total RNA was isolated from liver tissues and cultured cells using the Total RNA Isolation Kit (Yeason, China) following the manufacturer's protocol. Relative mRNA levels were quantified by real-time PCR and normalized to β-actin expression. The primer sequences used for amplification (Tsingke, Wuhan, China) are provided in **Supplementary Table S2**. For protein analysis, western blotting was conducted on both hepatic tissue samples and cultured cells to determine target protein expression. β-actin was applied as a loading control. Immunoblot band intensities were quantified using ImageJ/Fiji with identical background-subtraction and region-of-interest settings across samples. The detailed list of primary and secondary antibodies employed in the assays is presented in **Supplementary Table S3**.

### Immunohistochemical and immunofluorescence staining

Histological evaluation of liver tissues was performed using hematoxylin and eosin (H&E) staining. Immunohistochemistry (IHC) was carried out to detect the expression of PAK1 (21401-1-AP; Proteintech), Ly-6G (GB11229; Servicebio) and F4/80 (ab300421; Abcam). The staining intensity of IHC sections was independently graded by two experienced pathologists on six levels: 0 (no staining), 1 (weakly positive), 2 (weak-to-moderate positive), 3 (moderately positive), 4 (moderate-to-strong positive), and 5 (strongly positive). Apoptotic cells were identified by the TUNEL staining (PN0048; Pinuofei) according to the manufacturer's instructions. Images were captured with a fluorescence microscope (IX83, Olympus).

### RNA-seq and data processing

AML12 cell line was transduced to achieve PAK1 overexpression and then subjected to hypoxia reoxygenation (HR) treatment. Total RNA was extracted, and poly(A) mRNA was enriched using oligo(dT) magnetic beads. RNA-seq libraries were prepared with the NEBNext Ultra RNA Library Prep Kit for Illumina (New England Biolabs) following the manufacturer's instructions. Sequencing was performed on an Illumina platform by Guangke Ande Biotechnology Co., Ltd. Differential gene expression and statistical significance were analyzed using DESeq2. Genes were considered differentially expressed when the absolute log₂ fold change |log₂FC| exceeded 1.5 with a false discovery rate (FDR) < 0.05. Gene set variation analysis (GSVA) was performed on the normalized RNA-sequencing expression matrix using the GSVA package in R to estimate pathway activity in individual samples. GSVA scores for the selected gluconeogenesis-related gene sets were visualized as a heatmap.

### LC-MS/MS identification of PCK1 lactylation

Protein lysates were immunoprecipitated using beads pre-coupled to an anti-PCK1 antibody. The bead-bound immunoprecipitates were submitted without elution to Jingjie PTM BioLab (Hangzhou, China) for LC-MS/MS analysis. The enriched proteins were enzymatically digested, and the resulting peptides were analysed by LC-MS/MS. Candidate PCK1 lactylation sites were identified by database searching with lysine lactylation specified as a variable modification.

### Statistical analysis

Significance was defined at P < 0.05, with the following notation: P < 0.05 (*), P < 0.01 (**), P < 0.001 (***), and P < 0.0001 (****). All computations were performed in GraphPad Prism 9.0 (GraphPad Software, La Jolla, CA, USA). Continuous outcomes were summarized as mean ± standard deviation (SD), whereas categorical variables are reported as counts or frequencies. Whole-slide images were analyzed in QuPath (0.6.0 version) using a uniform pipeline with manually annotated regions of interest to exclude artifacts, followed by automated cell detection and intensity-based classification of positive cells using a predefined threshold. Positivity was reported as the percentage of positively stained cells among all detected cells within each region (averaged per case when multiple regions were analyzed). For approximately normally distributed data, two-group comparisons used unpaired, two-sided Student's t tests. Comparisons across multiple groups employed one-way ANOVA procedures. Data not conforming to normality were analyzed with the Wilcoxon test.

## Results

### PAK1 is activated during steatotic hepatic IR injury and is associated with reduced post-transplant liver injury

To define how PAK1 responds to steatotic hepatic IR injury, we examined experimental models and human liver grafts. Hepatic PAK1 mRNA was higher in high-fat diet-fed mice after IR than after sham surgery (Figure 1A). In representative immunoblots of AML12 cells, H/R increased phosphorylated PAK1 (P-PAK1) and total PAK1 abundance (Figure 1B). Mouse liver immunoblotting and immunohistochemistry likewise showed increased PAK1 activation and expression after IR (Figure 1C-F). In human graft biopsies, reperfusion markedly increased P-PAK1, whereas total PAK1 showed no significant change (Figure 1G, H). We next related graft PAK1 IHC scores to postoperative outcomes in 74 recipients. PAK1 scores were inversely correlated with serum ALT and AST levels on postoperative day 3, and both markers were lower in the PAK1-high group than in the PAK1-low group (Figure 1I-L). Representative IHC images illustrated the two expression categories (Figure 1M). The PAK1-high group also showed better graft survival than the PAK1-low group (log-rank P = 0.003; Figure 1N). EAD occurred in 30% and 35% of the PAK1-high and PAK1-low groups, respectively (P = 0.63; Figure S1A).

### Hepatocyte-specific PAK1 overexpression ameliorate hepatocellular injury and the inflammatory responses during IR injury

To assess the functional role of PAK1, we generated stable PAK1-overexpressing (PAK1-OE) AML12 cells and exposed them to OA plus H/R. PAK1 overexpression increased Bcl-2 and reduced Bax and cleaved caspase-3 in representative immunoblots (Figure 2A; Figure S1B, C). It also reduced ROS fluorescence and yielded flow-cytometry profiles consistent with reduced apoptosis (Figure 2B, C). We then used an AAV8 vector to overexpress PAK1 in mouse liver. PAK1 overexpression did not materially alter serum TG, TC, and LDL-C levels. Oil Red O staining also showed no obvious difference in hepatic lipid accumulation (Figure S1D-G). After 90 min of ischemia and 6 h of reperfusion, PAK1-OE mice had lower serum ALT, AST, and IL-6 levels than control mice (Figure 2D-F). Serum IL-1β and TNF-α levels were also reduced (Figure S1H, I). Hepatic IL-6, IL-1β, TNF-α, and CXCL10 transcripts showed concordant decreases (Figure S1J-M). In IR livers, PAK1 overexpression increased PAK1 and P-PAK1, while reducing Bax and cleaved caspase-3 (Figure 2G, H). TUNEL, H&E, F4/80, and Ly6G staining showed less apoptosis, tissue injury, macrophage accumulation, and neutrophil accumulation in PAK1-OE livers (Figure 2I-M).

### Hepatocyte-specific PAK1 knockdown exacerbates apoptosis and inflammatory responses after IR injury

Reciprocal loss-of-function experiments were performed in AML12 cells and mice. In OA-treated AML12 cells, PAK1 knockdown reduced PAK1 and Bcl-2 levels, while increasing Bax and cleaved caspase-3 in representative immunoblots (Figure 3A; Figure S2A, B). PAK1 knockdown also enhanced ROS fluorescence and altered the flow-cytometry quadrant distributions (Figure 3B, C). In mice, AAV8-shPAK1 increased serum ALT, AST, and IL-6 after IR (Figure 3D-F). Serum TNF-α was also increased (Figure S2C). In IR livers, PAK1 knockdown reduced P-PAK1 and Bcl-2 and increased cleaved caspase-3 (Figure 3G, H). TUNEL, H&E, F4/80, and Ly6G staining showed more severe apoptosis, tissue injury, macrophage accumulation, and neutrophil accumulation after PAK1 knockdown (Figure 3I-M). Pharmacological PAK inhibition with FRAX597 recapitulated these effects. FRAX597 increased serum ALT and AST (Figure S2D, E), reduced P-PAK1 and Bcl-2, and increased Bax and cleaved caspase-3 without changing total PAK1 (Figure S2F, G). It also increased hepatic IL-6, IL-1β, and TNF-α transcripts (Figure S2H-J). In PAK1-OE AML12 cells, FRAX597 blunted the anti-apoptotic protein profile associated with PAK1 overexpression (Figure S2K). TUNEL and histological analyses further showed increased apoptosis and inflammatory-cell accumulation after FRAX597 treatment (Figure S2L, M).

### PAK1 promotes PCK1 lactylation and gluconeogenic reprogramming during steatotic hepatic IR injury

To investigate the metabolic program associated with PAK1, we performed RNA sequencing in control and PAK1-OE AML12 cells after OA plus H/R. GSVA showed higher scores for GO positive regulation of gluconeogenesis and KEGG glycolysis/gluconeogenesis in PAK1-OE cells (Figure 4A, B). Concordant increases were observed for Reactome gluconeogenesis and WikiPathways glycolysis/gluconeogenesis (Figure S3A, B). A pathway-score heatmap showed the same group-level pattern across all four gene sets (Figure 4C). Gluconeogenesis can change non-carbohydrate carbon substrates, such as lactate, amino acids, into glucose to maintain energy demands during fasted and stressed conditions^16^. So, Periodic acid-Schiff (PAS) staining was performed using the previously described human liver tissue microarray samples. The results showed that the glycogen staining level was significantly increased in the PAK1 high-expression group (Figure 4D, E). Furthermore, PAS staining was also performed on liver tissues from PAK1-OE mice, which showed a significantly higher level of glycogen deposition compared with the control group (Figure 4F). Gluconeogenesis is primarily regulated by three rate-limiting enzymes responsible for the irreversible steps of the pathway: phosphoenolpyruvate carboxykinase (PCK1), fructose-1,6-bisphosphatase (FBP1), and glucose-6-phosphatase (G6PC)^17^. Sodium lactate and PAK1 overexpression each increased PCK1 activity and glucose production in mouse liver and AML12 cells (Figure 4G-J). Then, we treated the hepatic IR mice with 3-MPA, a well-studied PCK1 inhibitor^18^. Both the serum analysis for ALT, AST, western blot for apoptotic proteins (Bax and Bcl-2) and RT-qPCR for inflammatory cytokines (IL-6, IL-1β and TNF-α) showed that inhibiting PCK1 activity by 3-MPA aggravated hepatic IR injury (Figure S3C-H). Further H&E staining, F4/80 and Ly-6G immunohistochemistry, as well as TUNEL fluorescence staining, also supported these findings (Figure S3I, J). As PCK1 was a carrier directly involved in glycometabolism and accumulating evidence indicated that PCK1 was subjected to multiple post-translational modifications (PTMs), which altered its enzymatic activity and stability^19^. We hypothesized that PCK1 may affected hepatic glycometabolism through lysine lactylation. Immunoprecipitation of PCK1 followed by pan-Kla immunoblotting showed a stronger PCK1 lactylation signal after PAK1 overexpression in cells and mouse liver (Figure 4K, L). Conversely, shPAK1 reduced the PCK1-associated pan-Kla signal under H/R (Figure 4M). LC-MS/MS detected a PCK1 peptide carrying lactylation at K547 and K551 (Figure S3K). Structural modeling predicted local conformational effects at both sites (Figure 4N; Figure S3L). In qualitative immunoblots, the K547R substitution most clearly a1ttenuated the anti-apoptotic protein pattern associated with wild-type PCK1. This observation was consistent with a functionally important role for K547 lactylation (Figure 4O).

### PAK1-mediated phosphorylation of ACSS2 drives the assembly of an ACSS2-KAT2A-PCK1 complex to promote PCK1 lactylation

We next investigated how PAK1 was linked to PCK1 lactylation. Reciprocal co-immunoprecipitation and confocal immunofluorescence showed an association and cytoplasmic colocalization between PCK1 and KAT2A (Figure 5A, B). Then, we treated the hepatic IR mice with MB-3, the KAT2A-specific chemical inhibitor^20^. In mice, pharmacological KAT2A inhibition with MB-3 increased serum ALT, AST, and TNF-α and increased hepatic inflammatory transcripts after IR (Figure S4A-F). MB-3 also increased Bax and cleaved caspase-3, while Bcl-2 did not change significantly (Figure S4G, H). TUNEL, H&E, F4/80, and Ly6G staining showed greater apoptosis, tissue injury, macrophage accumulation, and neutrophil accumulation after KAT2A inhibition (Figure S4I, J). We further overexpressed PAK1 and knocked down KAT2A in AML12 cells. After KAT2A knockdown, PCK1 lactylation was significantly reduced even in the presence of PAK1 overexpression, indicating that PAK1 promotes PCK1 lactylation primarily through KAT2A (Figure S4K).

It has been reported that ACSS2 acts as a bona fide lactyl-CoA synthetase, converting lactate into lactyl-CoA, which subsequently binds directly to KAT2A^21^. Co-IP, western blot and confocal immunofluorescence analyses have demonstrated the direct interaction between the Flag-KAT2A and HA-ACSS2 (Figure 5C-D). To assess the potential role of PAK1 in regulating ACSS2 through phosphorylation, we overexpressed PAK1 in AML12 cells and found that the phosphorylation level of ACSS2 was significantly increased (Figure 5E). Co-IP, western blot and confocal immunofluorescence analyses also demonstrated the direct interaction between the HA-ACSS2 and Flag-PAK1 (Figure 5F-G). The S659 and S267 sites of ACSS2 have been reported to affect its functional activity^22^, ^23^. Compared with wild-type ACSS2, the S267A variant produced the clearest reduction in Bcl-2, increase in Bax, and decrease in the global pan-Kla signal (Figure 5H). These qualitative data were consistent with a functionally important role for S267 in PAK1-associated ACSS2 regulation.

### Attenuation of IR injury by PAK1 agonist Fingolimod

Fingolimod (FTY720), an immunosuppressive drug, has been reported to have the potential to activate PAK1 and to exert protective effects against liver injury 24, 25. The chemical structure of FTY720 was shown in Figure 6A. In high-fat diet-fed mice, FTY720 lowered serum ALT, AST, and total bilirubin after hepatic IR (Figure 6B-D). FTY720 increased P-PAK1 and Bcl-2, while reducing total PAK1 and cleaved caspase-3 (Figure 6E, F). H&E, F4/80, Ly6G, and TUNEL analyses showed reduced tissue injury, macrophage accumulation, neutrophil accumulation, and apoptosis (Figure 6G, H; Figure S6A-C). PAS staining showed greater hepatic glycogen deposition after FTY720 treatment (Figure 6I). FTY720 also reduced malondialdehyde and increased superoxide dismutase and glutathione levels (Figure 6J-L). Treatment with the S1PR1-selective agonist SEW2871 also lowered serum liver-injury markers and inflammatory transcripts (Figure S5A-G). Representative immunoblots, TUNEL staining, and histology were qualitatively consistent with reduced apoptosis and neutrophil accumulation (Figure S5H-J). To test the contribution of hepatic PAK1, FTY720 was administered to AAV8-shPAK1 mice. PAK1 knockdown substantially weakened the FTY720-associated reductions in ALT and AST (Figure S6D, E). Apoptosis-related proteins and histological injury no longer differed between AAV8-shPAK1 mice receiving FTY720 and injured AAV8-NC controls receiving vehicle (Figure S6F-H). Our results demonstrated that FTY720 alleviate steatotic hepatic IR injury at least in part through the activation of PAK1, suggesting that FTY720 could serve as a potential therapeutic agent for steatotic hepatic IR injury.

## Discussion

Hepatic IR injury was a multifactorial and intricate pathological process that significantly influenced postoperative outcomes in liver transplantation and hepatectomy. Currently, an increasing number of steatotic donor livers have been used in clinical transplantation, and these grafts were more susceptible to IR injury. Despite extensive investigation into its underlying mechanisms and potential therapeutic approaches, clinically effective intervention remained limited. In this study, we found that the expression of PAK1 was upregulated after IR injury in patients and mice, and was associated with improved graft function and recipient survival following LT. Through gain- and loss-of-function experiments, we found that PAK1 knockout exacerbated IR-induced hepatic inflammatory response and hepatocyte apoptosis, while PAK1 overexpression produced protective effects. Mechanistically, PAK1 interacted directly with ACSS2 to mediate the S267 phosphorylation. The phosphorylated ACSS2 then recruited KAT2A to form the multiprotein complex that catalyzed PCK1 lactylation locally, therefore promoting gluconeogenesis. And the Fingolimod was found to protect against steatotic IR injury in the model effectively. These results suggest that PAK1 may be an important target for alleviating steatotic hepatic IR injury (Figure 7).

PAK1 was one of the serine/threonine kinases activated by Rho-family GTPases, including Ras-related C3 botulinum toxin substrate 1 (Rac1) and cell division control protein 42 homolog (Cdc42), has been widely studied as an oncogenic protein that was overexpression in many types of cancer^26^, ^27^. The upstream regulation of PAK1 during steatotic hepatic IR injury is multifactorial. In the present study, Rac1 inhibition reduced H/R induced PAK1 phosphorylation, suggesting that Rac1 contributes to PAK1 activation in hepatocytes under H/R stress. In addition, IR-related oxidative stress, cytoskeletal remodeling, inflammatory receptor signaling, and sphingolipid/sphingosine-1-phosphate (S1P) receptor pathways may converge on Rac1/Cdc42-PAK1 signaling^28^. Consistent with the role of PAK1 in IR injury, PAK1 has been shown to improve post-ischemic functional recovery in a cardiac IR injury model^29^. Therefore, the Rac1/Cdc42-PAK1 axis may represent an upstream signaling module that links stress sensing to the metabolic hepatoprotection, although the precise receptor-level trigger remains to be defined.

PAKs participated in the modulation of various inflammatory responses, and PAKs have been reported to play a critical role in hepatic IRI^30^, ^31^. P21-activated kinase 4 (PAK4) has been reported to be up-regulated in hepatic IR injury and its inhibition could alleviate inflammation and necrosis of hepatocytes through up-regulating the transcriptional activity of nuclear factor erythroid 2-related factor 2 (Nrf2)^8^, ^32^. And recently one study has reported that human amniotic epithelial cells alleviate ischemia-reperfusion injury of steatotic livers through mediating PAK1/AMPK-dependent autophagy^33^. In addition, neuregulin-1/PAK1 axis was found to attenuate hepatic IR injury of liver grafts with or without steatosis through increasing vascular endothelial growth factor-α and insulin growth factor-1 levels, respectively^34^. Interestingly, in cardiomyocytes subjected to IR injury, PAK1 improved cardiac contractility, accompanied by changes in the phosphorylation of troponin T and myosin light chain 2^29^. In our study, we also showed that PAK1 level was associated with improved graft function and recipient survival following LT. And, we found that PAK1 overexpression protect steatotic liver from IR injury, while PAK1 knockout aggravated IR-induced hepatic inflammatory response and hepatocyte apoptosis. Such results showed that PAK1 may participated in the repair process of steatotic liver injury.

As a key enzyme in gluconeogenesis, PCK1 has been reported to play an important role in the process of liver injury. Previous study examining gluconeogenic function in renal tissues have revealed a transient activation of this gene following ischemia injury. Consistent with this finding, PCK1 has been shown to be upregulated in the rat brain following ischemic stroke and in cultured neurons subjected to oxygen-glucose deprivation^35^. Moreover, clinical and animal experiments have indicated that defective glucose production and impaired lactate clearance were strongly correlated to poor outcomes and higher mortality in acute kidney injury^36^. Recently, one study has reported that the donor liver may initiate gluconeogenesis through the activation of the METTL3/m^6^A-PCK1 axis to reduce the loss of hepatocellular energy and foster glycogen storage for use in the early postoperative process, thus attenuating liver injury^13^. However, the role of PCK1 in hepatic IR injury of steatotic livers has not yet been elucidated.

In 2019, Yingming Zhao and his team demonstrated the existence of histone lysine lactylation (Kla) and identified it as a novel type of post-translational epigenetic modification derived from lactate^10^. It could regulate gene expression directly. In recent times, non-histone protein lactylation has been increasingly reported to play an important role in modulating protein function and participating in diverse cellular regulatory processes^37^, ^38^. ACSS2 was a protein that synthesized acetyl-CoA to produce acetyl-CoA from acetate, which was essential for maintaining histone H3/H4 acetylation and sustaining transcription activity during metabolic stress^39^. ACSS2 has also been reported to act as a β-hydroxybutyryl-CoA synthetase in cooperation with KAT7, sensing β-hydroxybutyrate and converting it into BHB-CoA to drive H3K9 Kbhb, thereby upregulating genes involved in metabolism^40^. These findings collectively define ACSS2 as a “metabolic write” that sensed diverse carbon metabolites and locally converted them into reactive acyl-CoA donors for protein modification. The histone acetyltransferase KAT2A was increasingly recognized as a critical epigenetic regulator that links acyl-CoA-derived metabolites to chromatin modifications, thereby influencing transcriptional activity in development, metabolism and cancer. Recent studies also reported that it was capable of mediating non-acetyl acylations, shaping chromatin landscapes, and sustaining pathological states^41^, ^42^. Consistent with the above findings, in our study, we found that PAK1 could integrate directly with ACSS2 to mediate its phosphorylation. The phosphorylated ACSS2 then recruited KAT2A directly to form the multiprotein complex that promoted PCK1 lactylation locally, therefore promoting gluconeogenesis.

We acknowledged that the current study exerts some limitations. Firstly, We used an AAV8-based lentiviral system to specifically knock down PAK1 expression in the liver and demonstrated that this exacerbated hepatic IR injury in steatotic liver. Validation using a small-molecule PAK1 inhibitor yielded consistent results. However, further confirmation in liver-specific PAK1 knockout mice was not performed. Secondly, we demonstrated that lactylation of PCK1 affected its protein function through a series of experiments, however, the specific lactylation sites have not yet been identified. Third, we only focused on KAT2A as the lactyltransferase. But in our study, KAT2A showed reproducible association with PCK1 in pull-down experiments, and KAT2A inhibition or knockdown reduced the PAK1-driven lactylation phenotype. Nevertheless, other acyltransferases may participate in PCK1 lactylation under different metabolic stresses, and broader enzyme-screening studies will be needed to fully define the lactyltransferase landscape.

## Conclusion

In summary, our study demonstrated that PAK1 was a critical protective factor in hepatic IR injury for steatotic liver. Mechanistically, PAK1 could directly bind ACSS2 and facilitate its phosphorylation at S267. The phosphorylated ACSS2 subsequently recruited KAT2A, leading to the assembly of a multiprotein complex responsible for the local lactylation of PCK1, thereby enhancing gluconeogenic activity. This metabolic adaptation mitigated ROS accumulation and reinforced cellular energy homeostasis. Moreover, Fingolimod effectively alleviated steatotic IR injury. These findings open new therapeutic avenues for steatotic IR injury and underscore the potential of PAK1 as therapeutic target in steatotic liver injury.

## Supplementary Material

Supplementary figures and tables.

## Figures and Tables

**Figure 1:**
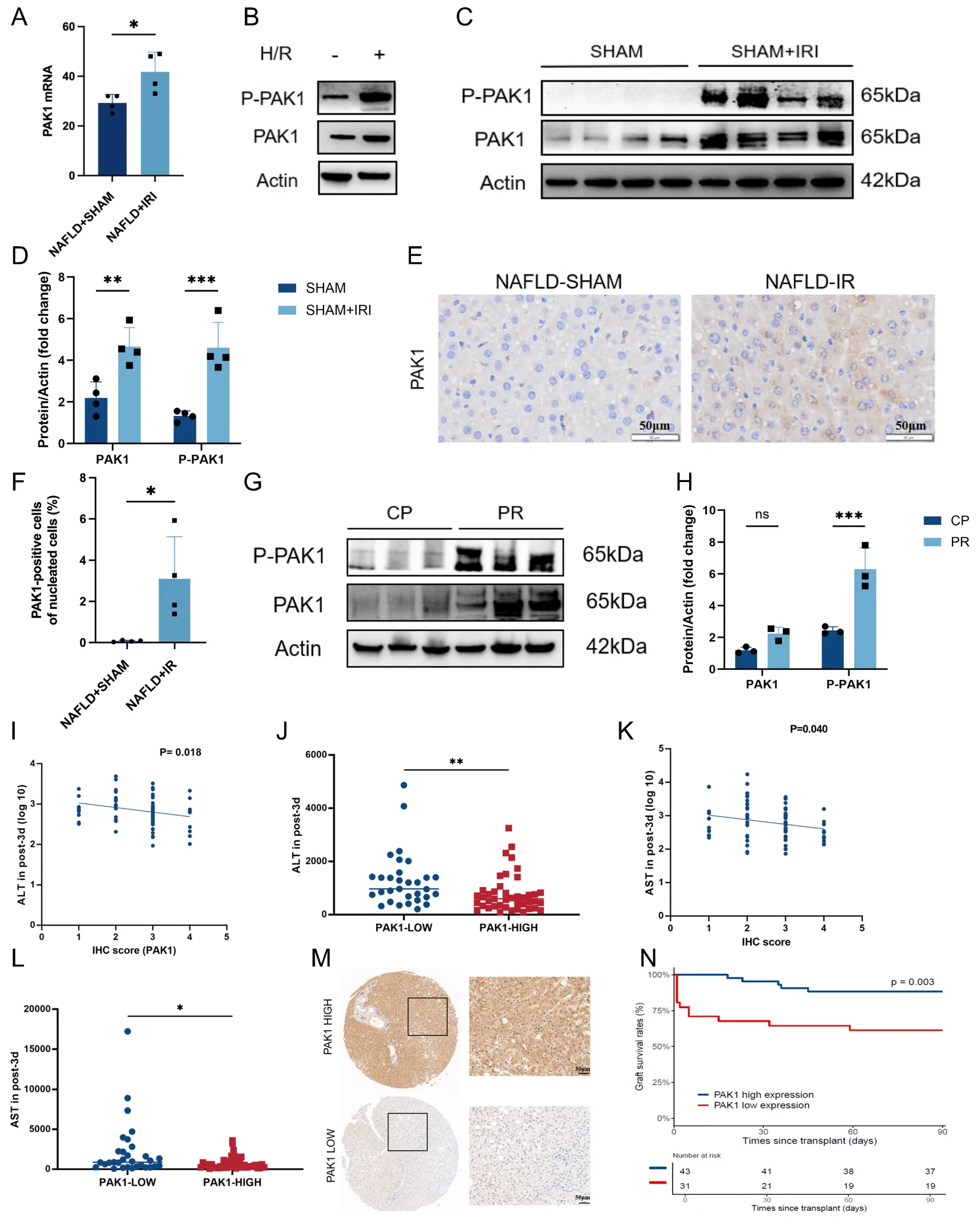
** PAK1 is activated during steatotic hepatic ischemia-reperfusion injury and is associated with reduced post-transplant liver injury.** (A) Hepatic PAK1 mRNA in high-fat diet-fed mice after sham surgery or IR (n = 4 mice per group). (B) Representative immunoblot of P-PAK1 and PAK1 in AML12 cells with or without H/R; actin was the loading control. (C, D) Representative immunoblots and quantification of P-PAK1 and PAK1 in mouse livers after sham surgery or IR (n = 4 mice per group). (E, F) Representative PAK1 IHC and quantification in steatotic mouse livers after sham surgery or IR (n = 4 mice per group). Scale bar, 50 µm. (G, H) Representative immunoblots and quantification of P-PAK1 and PAK1 in human graft biopsies obtained during cold preservation (CP) and after reperfusion (PR) (n = 3 grafts per phase). (I) Association between graft PAK1 IHC score and log10-transformed serum ALT on postoperative day 3 (n = 74 recipients). (J) Serum ALT on postoperative day 3 in the PAK1-low (n = 31) and PAK1-high (n = 43) groups. (K) Association between graft PAK1 IHC score and log10-transformed serum AST on postoperative day 3. (n = 74 recipients). (L) Serum AST on postoperative day 3 in the PAK1-low (n = 31) and PAK1-high (n = 43) groups. (M) Representative graft IHC overview images and enlargements of the boxed regions, showing low and high PAK1 expression. Scale bar, 50 µm. (N) Kaplan-Meier graft survival analysis stratified by PAK1 expression (PAK1-high, n = 43; PAK1-low, n = 31). One *in vivo* experiment using the indicated numbers of mice. Data are shown as mean ± SD; Student's t test for A and F. Two-way ANOVA for D and H. Survival curves were compared by the log-rank test. n.s. P > 0.05; *P < 0.05; **P < 0.01; ***P < 0.001. PAK1, p21-activated kinase 1; NAFLD, nonalcoholic fatty liver disease; IRI, ischemia reperfusion injury; H/R, hypoxia/reoxygenation; CP, cold preservation; PR, postreperfusion; IHC, immunohistochemistry; ALT, alanine aminotransferase; AST, aspartate aminotransferase; EAD, early allograft dysfunction.

**Figure 2:**
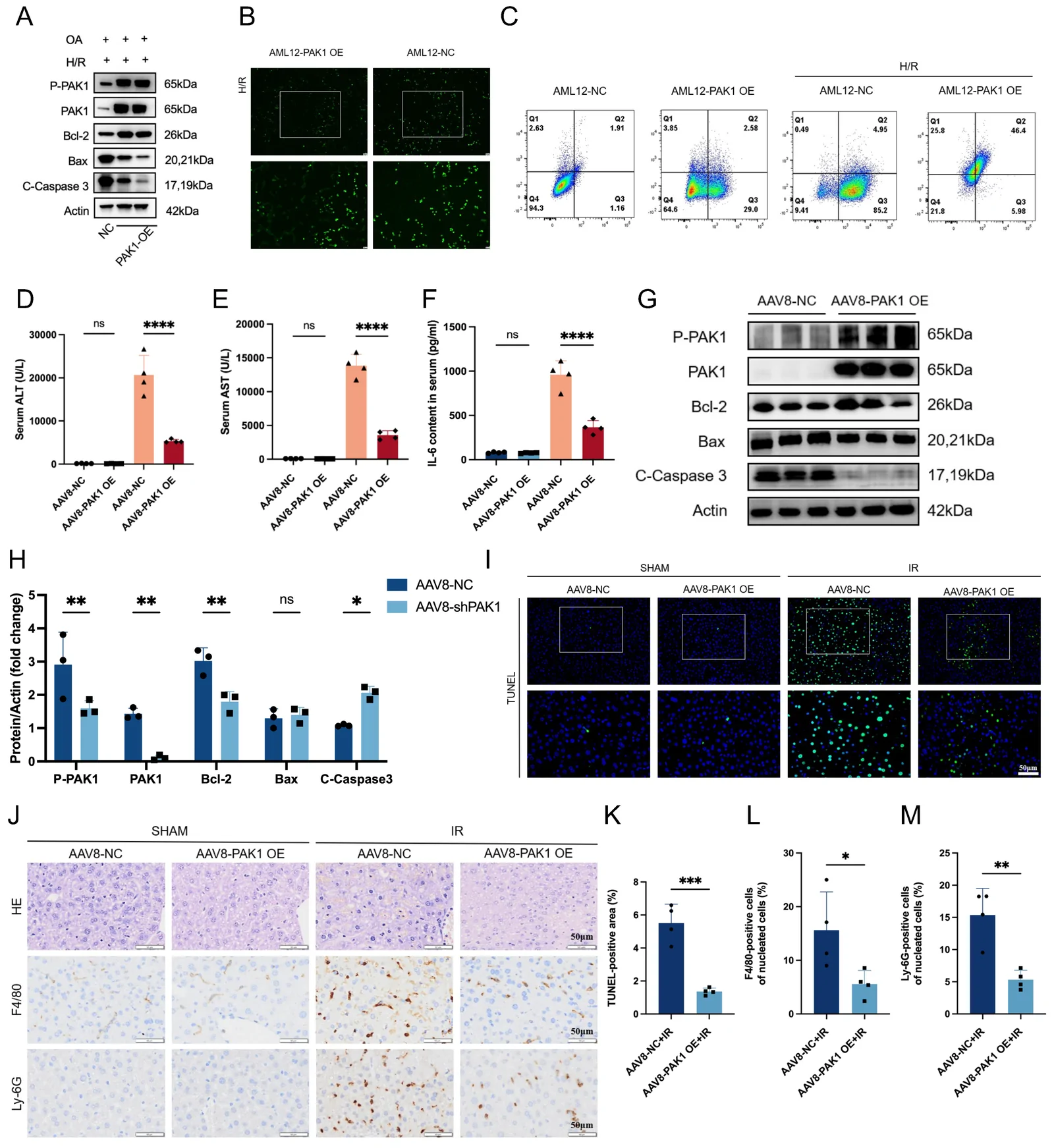
** Hepatocyte PAK1 overexpression attenuates apoptosis and inflammatory responses during hepatic ischemia-reperfusion.** (A) Representative immunoblot of P-PAK1, PAK1, Bcl-2, Bax, and cleaved caspase-3 in OA-treated AML12-NC and AML12-PAK1-OE cells after H/R; actin was the loading control. (B) Representative reactive oxygen species fluorescence in AML12-NC and AML12-PAK1-OE cells after H/R, shown as overview images and enlargements of the boxed regions. (C) Representative flow-cytometry profiles of AML12-NC and AML12-PAK1-OE cells under basal conditions and after H/R. (D-F) Serum ALT, AST, and IL-6 in AAV8-NC and AAV8-PAK1-OE mice after sham surgery or IR (n = 4 mice per group). (G, H) Representative liver immunoblots and quantification of P-PAK1, PAK1, Bcl-2, Bax, and cleaved caspase-3 after IR (n = 3 mice per group); actin was the loading control. (I) Representative TUNEL staining of liver sections after sham surgery or IR, shown as overview images and enlargements of the boxed regions (n = 4 mice per group). Scale bar, 50 µm. (J) Representative H&E, F4/80, and Ly6G staining after sham surgery or IR (n = 4 mice per group). Scale bar, 50 µm. (K-M) Quantification of TUNEL-positive area, F4/80-positive cells, and Ly6G-positive cells in IR livers (n = 4 mice per group). Three independent cell experiments for A-C, one *in vivo* experiment using the indicated numbers of biologically independent mice. Data are mean ± SD. One-way ANOVA was used for D-F, two-way ANOVA for H, and unpaired two-sided Student's t tests for K-M. ns, not significant; *P < 0.05; **P < 0.01; ***P < 0.001; ****P < 0.0001. OA, oleic acid; H/R, hypoxia/reoxygenation; IR, ischemia-reperfusion; AAV8, adeno-associated virus 8; OE, overexpression; NC, negative control; TUNEL, terminal deoxynucleotidyl transferase dUTP nick-end labeling; H&E, hematoxylin and eosin; ROS, reactive oxygen species; ALT, alanine aminotransferase; AST, aspartate aminotransferase; IL-6, interleukin-6.

**Figure 3:**
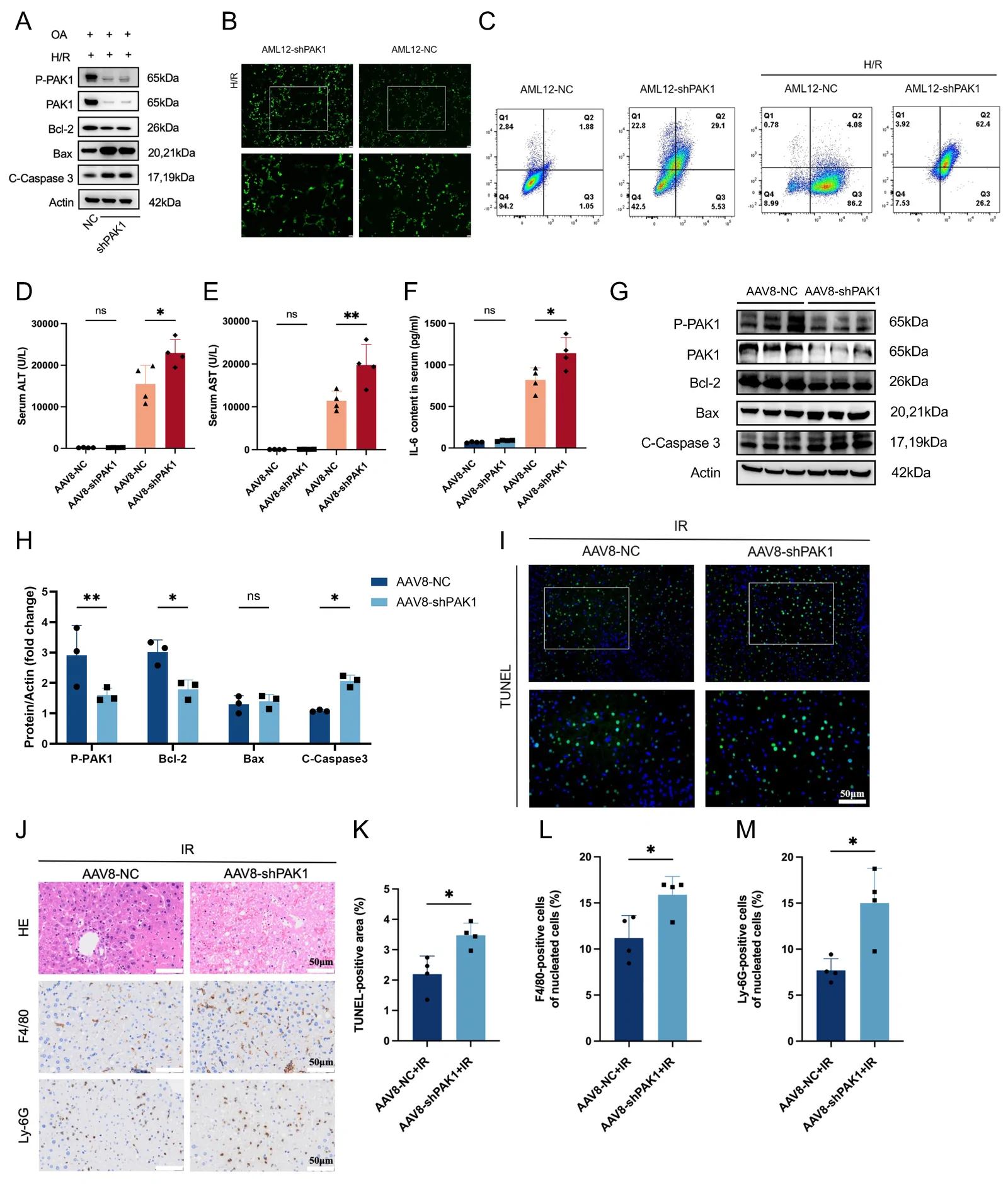
** Hepatic PAK1 knockdown aggravates hepatocellular apoptosis and inflammation during hepatic ischemia-reperfusion.** (A) Representative immunoblot of PAK1, Bcl-2, Bax, and cleaved caspase-3 in OA-treated AML12-NC and AML12-shPAK1 cells after H/R; actin was the loading control. (B) Representative reactive oxygen species fluorescence in AML12-NC and AML12-shPAK1 cells after H/R, shown as overview images and enlargements of the boxed regions. (C) Representative flow-cytometry profiles of AML12-NC and AML12-shPAK1 cells under basal conditions and after H/R. (D-F) Serum ALT, AST, and IL-6 in AAV8-NC and AAV8-shPAK1 mice after sham surgery or IR (n = 4 mice per group). (G, H) Representative liver immunoblots and quantification of P-PAK1, PAK1, Bcl-2, Bax, and cleaved caspase-3 after IR (n = 3 mice per group); actin was the loading control. (I) Representative TUNEL staining of IR liver sections from AAV8-NC and AAV8-shPAK1 mice, shown as overview images and enlargements of the boxed regions (n = 4 mice per group). Scale bar, 50 µm. (J) Representative H&E, F4/80, and Ly6G staining of IR liver sections (n = 4 mice per group). Scale bar, 50 µm. (K-M) Quantification of TUNEL-positive area, F4/80-positive cells, and Ly6G-positive cells in IR livers (n = 4 mice per group). Three independent cell experiments for A-C, one *in vivo* experiment using the indicated numbers of biologically independent mice. Data are mean ± SD. One-way ANOVA was used for D-F, two-way ANOVA for H, and unpaired two-sided Student's t tests for K-M. ns, not significant; *P < 0.05; **P < 0.01. OA, oleic acid; H/R, hypoxia/reoxygenation; IR, ischemia-reperfusion; AAV8, adeno-associated virus 8; shPAK1, short hairpin RNA targeting PAK1; NC, negative control; TUNEL, terminal deoxynucleotidyl transferase dUTP nick-end labeling; H&E, hematoxylin and eosin; ROS, reactive oxygen species; ALT, alanine aminotransferase; AST, aspartate aminotransferase; IL-6, interleukin-6.

**Figure 4:**
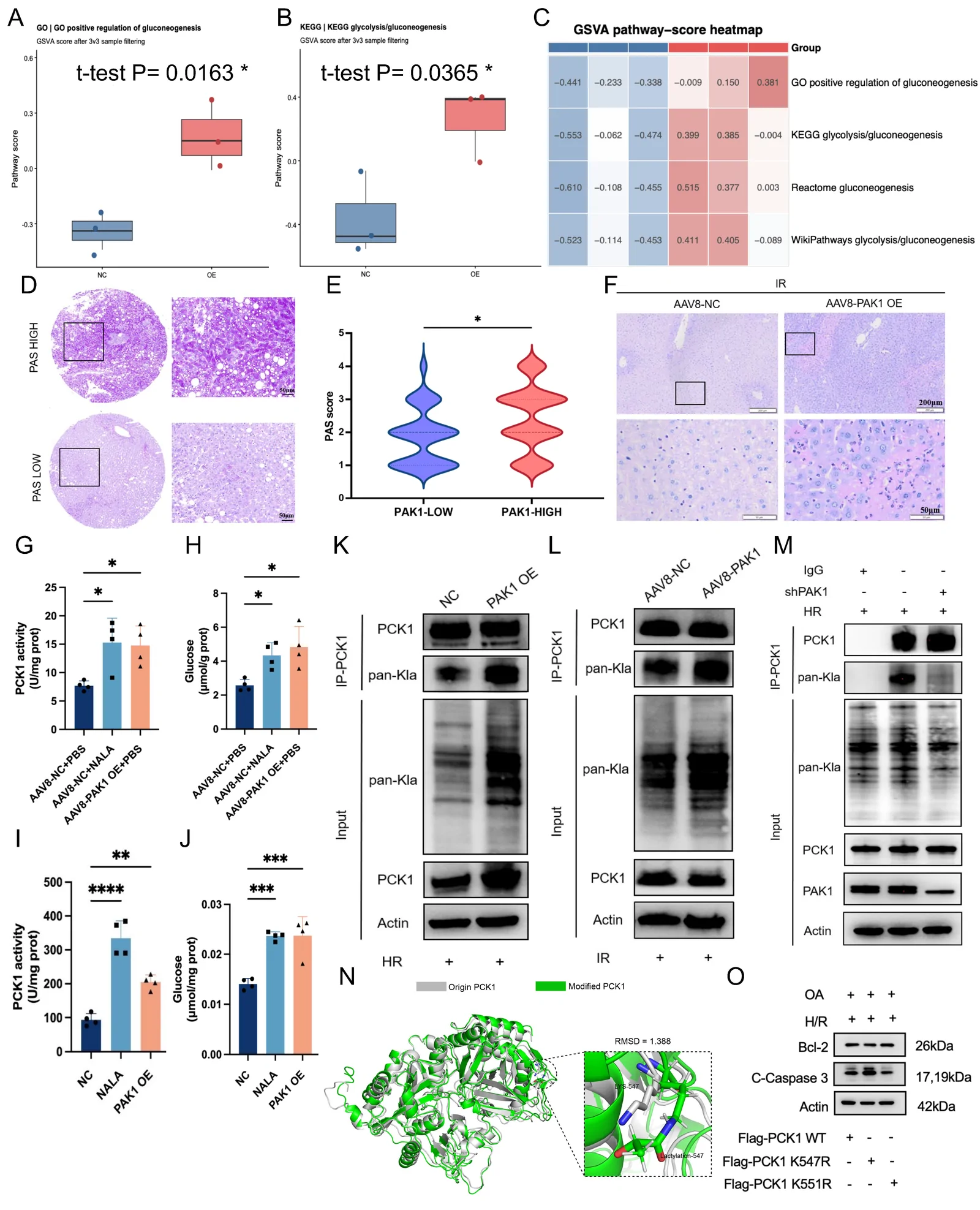
** PAK1 promotes PCK1 lactylation and gluconeogenic reprogramming during steatotic hepatic ischemia-reperfusion.** (A, B) GSVA pathway scores for GO positive regulation of gluconeogenesis and KEGG glycolysis/gluconeogenesis in OA-treated control and PAK1-OE AML12 cells after H/R (n = 3 samples per group). (C) GSVA pathway-score heatmap for four gluconeogenesis-related gene sets across the same samples. (D) Representative PAS staining of human graft sections with high or low PAS signals. Scale bar, 50 µm. (E) PAS scores in the PAK1-low and PAK1-high graft groups. (F) Representative PAS staining of IR livers from AAV8-NC and AAV8-PAK1-OE mice. Scale bars, 200 µm and 50 µm. (G, H) Hepatic PCK1 activity and glucose production in AAV8-NC+PBS, AAV8-NC+sodium lactate, and AAV8-PAK1-OE+PBS mice after IR (n = 4 mice per group). (I, J) PCK1 activity and glucose production in AML12 cells treated with control, sodium lactate, or PAK1 overexpression (n = 4 samples per group). (K) PCK1 immunoprecipitation followed by pan-Kla immunoblotting in control and PAK1-OE AML12 cells after H/R. (L) PCK1 immunoprecipitation and pan-Kla immunoblotting in IR livers from AAV8-NC and AAV8-PAK1-OE mice. (M) PCK1 immunoprecipitation and pan-Kla immunoblotting in control and shPAK1 AML12 cells after H/R; IgG was the negative control. (N) Structural superposition of native and K547-lactylated PCK1, with the modified region enlarged (RMSD = 1.388). (O) Representative Bcl-2 and cleaved caspase-3 immunoblots in OA-treated, H/R-exposed cells expressing Flag-PCK1 wild type, K547R, or K551R; actin was the loading control. One RNA-seq/GSVA analysis using n = 3 samples per group. Bar-graph data are mean ± SD. Unpaired two-sided Student's t tests were used for A, B, and E, and one-way ANOVA for G-J. *P < 0.05; **P < 0.01; ***P < 0.001; ****P < 0.0001. GSVA, gene set variation analysis; GO, Gene Ontology; KEGG, Kyoto Encyclopedia of Genes and Genomes; PAS, periodic acid-Schiff; PCK1, phosphoenolpyruvate carboxykinase 1; Kla, lysine lactylation; pan-Kla, pan-lysine lactylation; IP, immunoprecipitation; RMSD, root-mean-square deviation.

**Figure 5:**
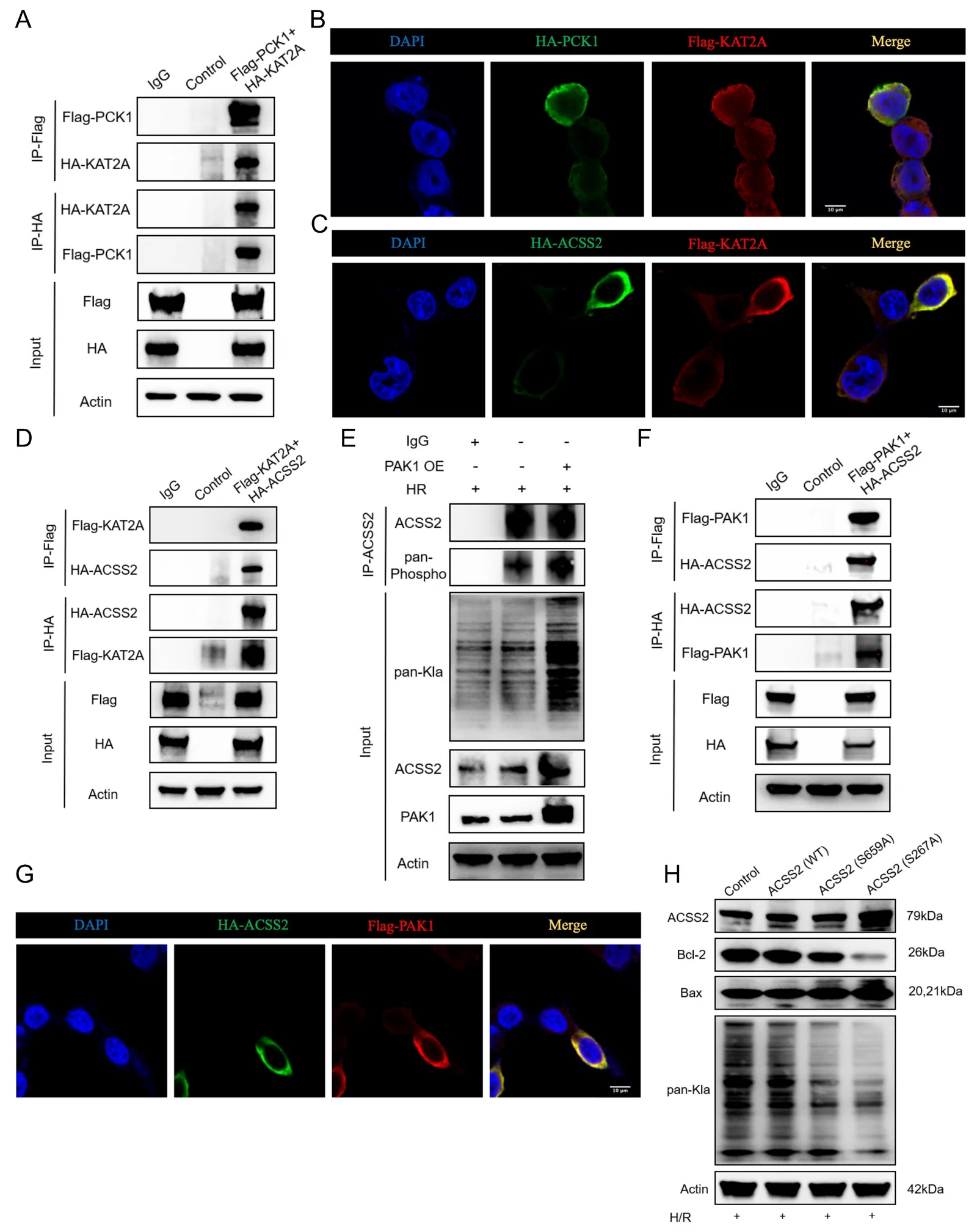
** PAK1-dependent phosphorylation of ACSS2 facilitates ACSS2-KAT2A-PCK1 complex formation, thereby promoting PCK1 lactylation.** (A) Reciprocal co-immunoprecipitation of Flag-PCK1 and HA-KAT2A; IgG and input controls are shown. (B) Representative confocal images of HA-PCK1 (green) and Flag-KAT2A (red), with DAPI nuclear counterstaining (blue). Scale bar, 10 µm. (C) Representative confocal images of HA-ACSS2 (green) and Flag-KAT2A (red). Scale bar, 10 µm. (D) Reciprocal co-immunoprecipitation of Flag-KAT2A and HA-ACSS2; IgG and input controls are shown. (E) ACSS2 immunoprecipitation followed by pan-phospho immunoblotting in control and PAK1-OE cells after H/R; IgG and input controls are shown. (F) Reciprocal co-immunoprecipitation of Flag-PAK1 and HA-ACSS2; IgG and input controls are shown. (G) Representative confocal images of HA-ACSS2 (green) and Flag-PAK1 (red). Scale bar, 10 µm. (H) Representative immunoblots of ACSS2, Bcl-2, Bax, and pan-Kla in control cells and cells expressing ACSS2 wild type, S659A, or S267A after OA treatment and H/R; actin was the loading control. PAK1, p21-activated kinase 1; ACSS2, acyl-CoA synthetase short-chain family member 2; KAT2A, lysine acetyltransferase 2A; PCK1, phosphoenolpyruvate carboxykinase 1; Kla, lysine lactylation; IP, immunoprecipitation; H/R, hypoxia/reoxygenation; WT, wild type.

**Figure 6:**
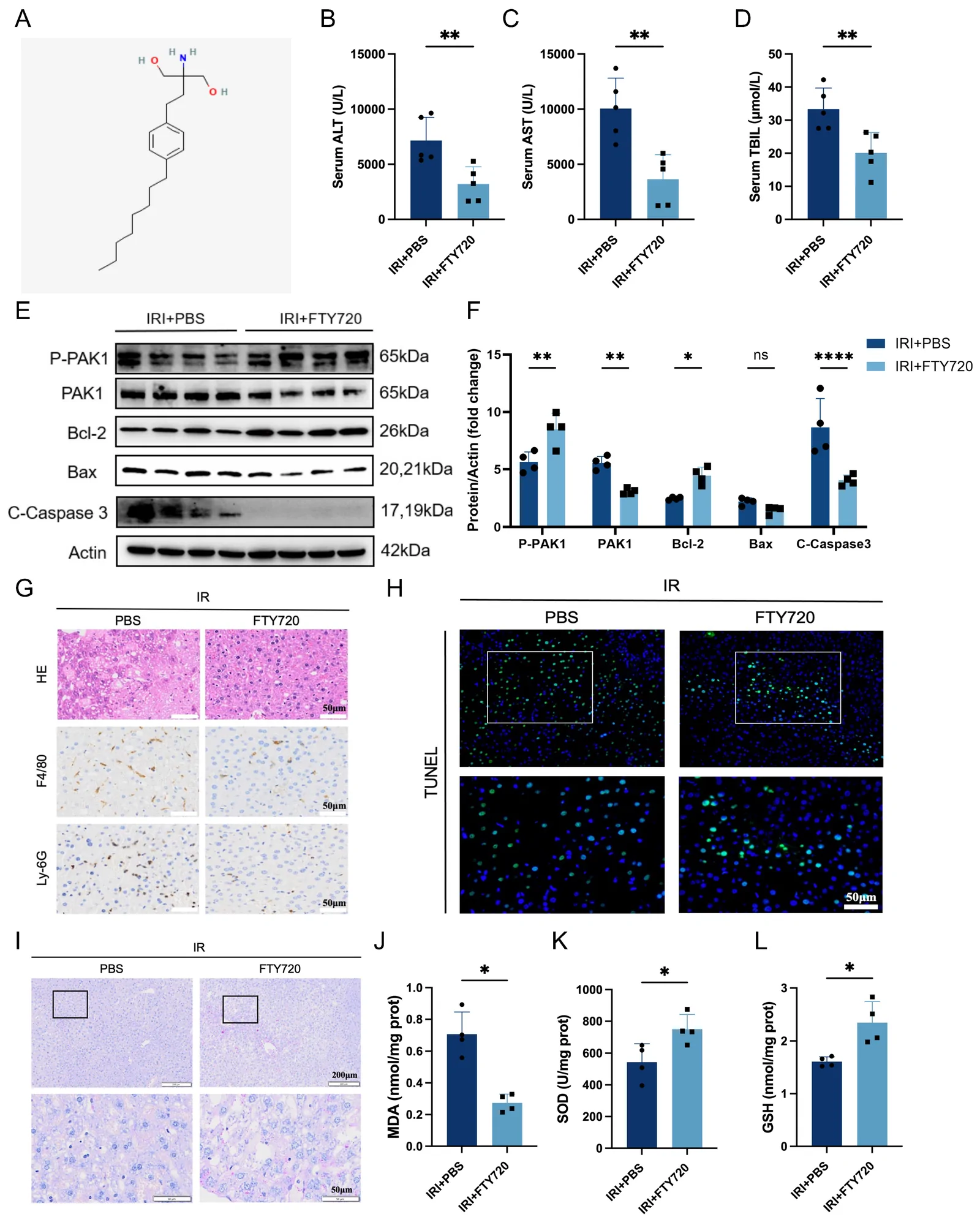
** FTY720 mitigates steatotic hepatic ischemia-reperfusion injury by suppressing apoptosis, inflammation, and oxidative stress.** (A) Chemical structure of FTY720. (B-D) Serum ALT, AST, and total bilirubin in mice after hepatic IR with vehicle or FTY720 treatment (n = 5 mice per group). (E, F) Representative liver immunoblots and quantification of P-PAK1, PAK1, Bcl-2, Bax, and cleaved caspase-3 after IR with vehicle or FTY720 (n = 4 mice per group); actin was the loading control. (G) Representative H&E, F4/80, and Ly6G staining after IR with vehicle or FTY720 (n = 4 mice per group). Scale bar, 50 µm. (H) Representative TUNEL staining after IR with vehicle or FTY720 (n = 4 mice per group). Scale bar, 50 µm. (I) Representative PAS staining after IR with vehicle or FTY720. Scale bars, 200 µm and 50 µm. (J-L) Hepatic malondialdehyde, superoxide dismutase, and glutathione levels after IR with vehicle or FTY720 (n = 4 mice per group). One *in vivo* experiment using the indicated numbers of biologically independent mice. Data are mean ± SD. Unpaired two-sided Student's t tests were used for B-D and J-L, and two-way ANOVA for F. ns, not significant; *P < 0.05; **P < 0.01; ****P < 0.0001. FTY720, fingolimod; IR, ischemia-reperfusion; ALT, alanine aminotransferase; AST, aspartate aminotransferase; PAK1, p21-activated kinase 1; P-PAK1, phosphorylated PAK1; PAS, periodic acid-Schiff.

**Figure 7:**
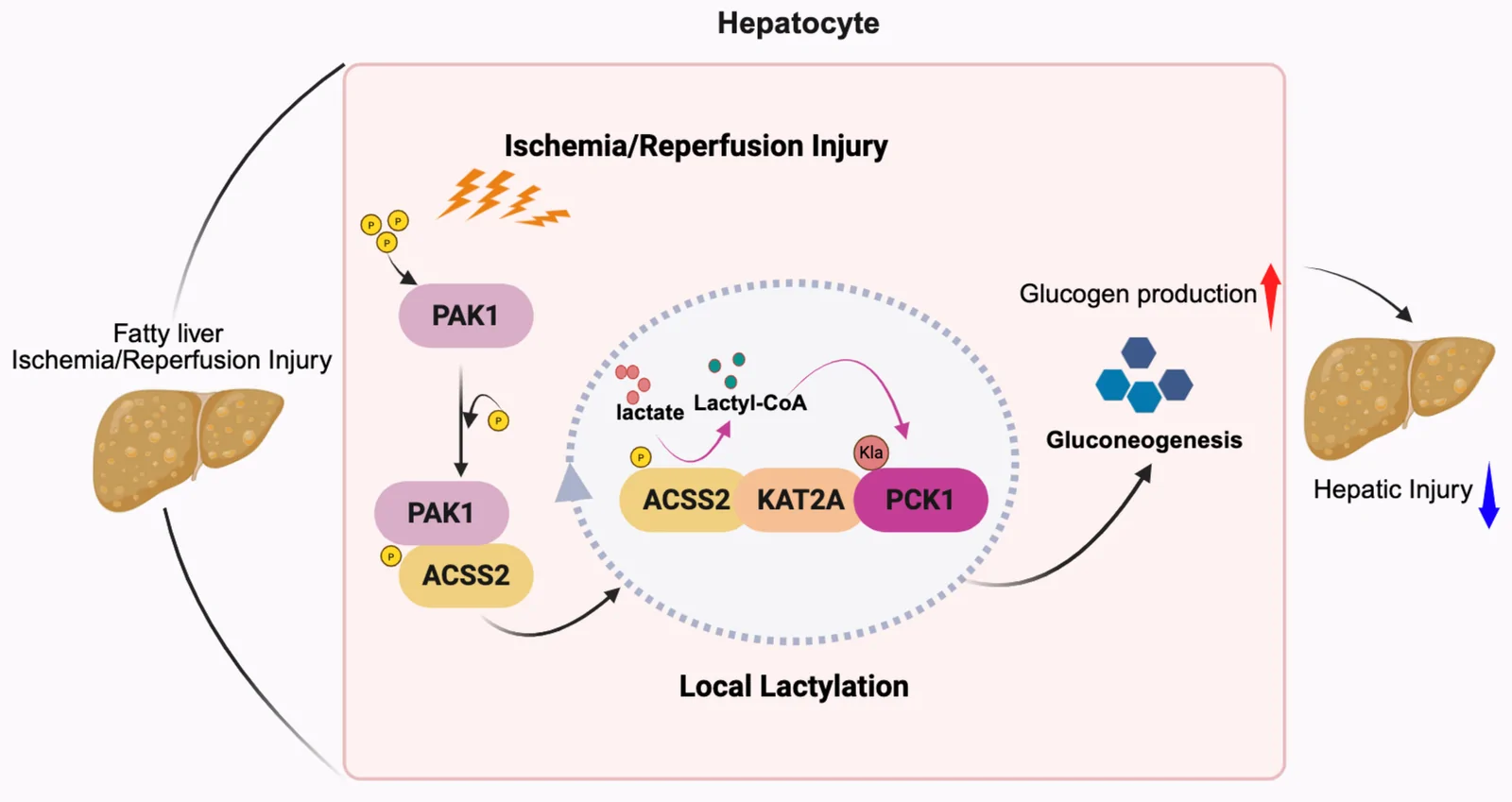
** Proposed model linking PAK1-ACSS2-KAT2A signaling to PCK1 lactylation and hepatoprotection.** Schematic illustrating that PAK1 interacts with ACSS2 and cooperates with KAT2A to facilitate lactate-derived lactyl-CoA generation and local protein lactylation, thereby enhancing PCK1 lactylation and gluconeogenesis/glycogen production and ultimately alleviating hepatic injury in fatty liver IR injury. PAK1, p21-activated kinase 1; ACSS2, acyl-CoA synthetase short-chain family member 2; KAT2A, lysine acetyltransferase 2A; PCK1, phosphoenolpyruvate carboxykinase 1; IR, ischemia reperfusion.

## Data Availability

All data generated or analyzed during this study are included in this published article.

## Abbreviations

PAK1: p21-activated kinase 1
P-PAK1: phosphorylated PAK1
AAV8: adeno-associated virus 8
ACSS2: acyl-CoA synthetase short-chain family member 2
ALT: alanine aminotransferase
AST: aspartate aminotransferase
CMV: cytomegalovirus immediate-early
CP: cold preservation
PR: postreperfusion
EAD: early allograft dysfunction
ELISA: enzyme-linked immunosorbent assay
FDR: false discovery rate
FTY720: fingolimod
GSEA: gene set enrichment analysis
GSH: glutathione
H&E: hematoxylin and eosin
H/R: hypoxia/reoxygenation
HFD: high-fat diet
IHC: immunohistochemistry
IF: immunofluorescence
IL-6: interleukin-6
IL-1β: Interleukin-1 beta
TNF-α: Tumor necrosis factor-alpha
CXCL10: C-X-C motif chemokine ligand 10
IP: immunoprecipitation
IRI: ischemia reperfusion injury
KAT2A: lysine acetyltransferase 2A
Kla: lysine lactylation
LT: liver transplantation
MAFLD: metabolic dysfunction-associated fatty liver disease
MDA: malondialdehyde
NAFLD: nonalcoholic fatty liver disease
NALA: sodium lactate
NC: negative control
NES: normalized enrichment score
Nrf2: nuclear factor erythroid 2-related factor 2
OA: oleic acid
OE: overexpression
OXSM: 3-oxoacyl-ACP synthase
PAK4: p21-activated kinase 4
PAKs: p21-activated kinases
PAS: periodic acid-Schiff
pan-Kla: pan-lysine lactylation
PCK1: phosphoenolpyruvate carboxykinase 1
qRT-PCR: quantitative reverse-transcription PCR
ROS: reactive oxygen species
SD: standard deviation
shPAK1: short hairpin RNA targeting PAK1
shRNA: short-hairpin RNA
SOD: superoxide dismutase
TBIL: total bilirubin
TC: total cholesterol
TG: triglycerides
TUNEL: terminal deoxynucleotidyl transferase dUTP nick end labeling
WT: wild type
