# Supplementary material for: PAK1 Promotes Gluconeogenesis Via the ACSS2/KAT2A/PCK1 Complex to Attenuate Steatotic Liver Ischemia-Reperfusion Injury
Source: Int J Biol Sci. 2026 Sep 11;22(15):8271–85. doi: 10.7150/ijbs.132770 (PMC13685614; doi:10.7150/ijbs.132770)
Supplement: Supplementary file 1 [file ijbsv22p8271s1.pdf]

Supplementary Data

Supplementary Figures

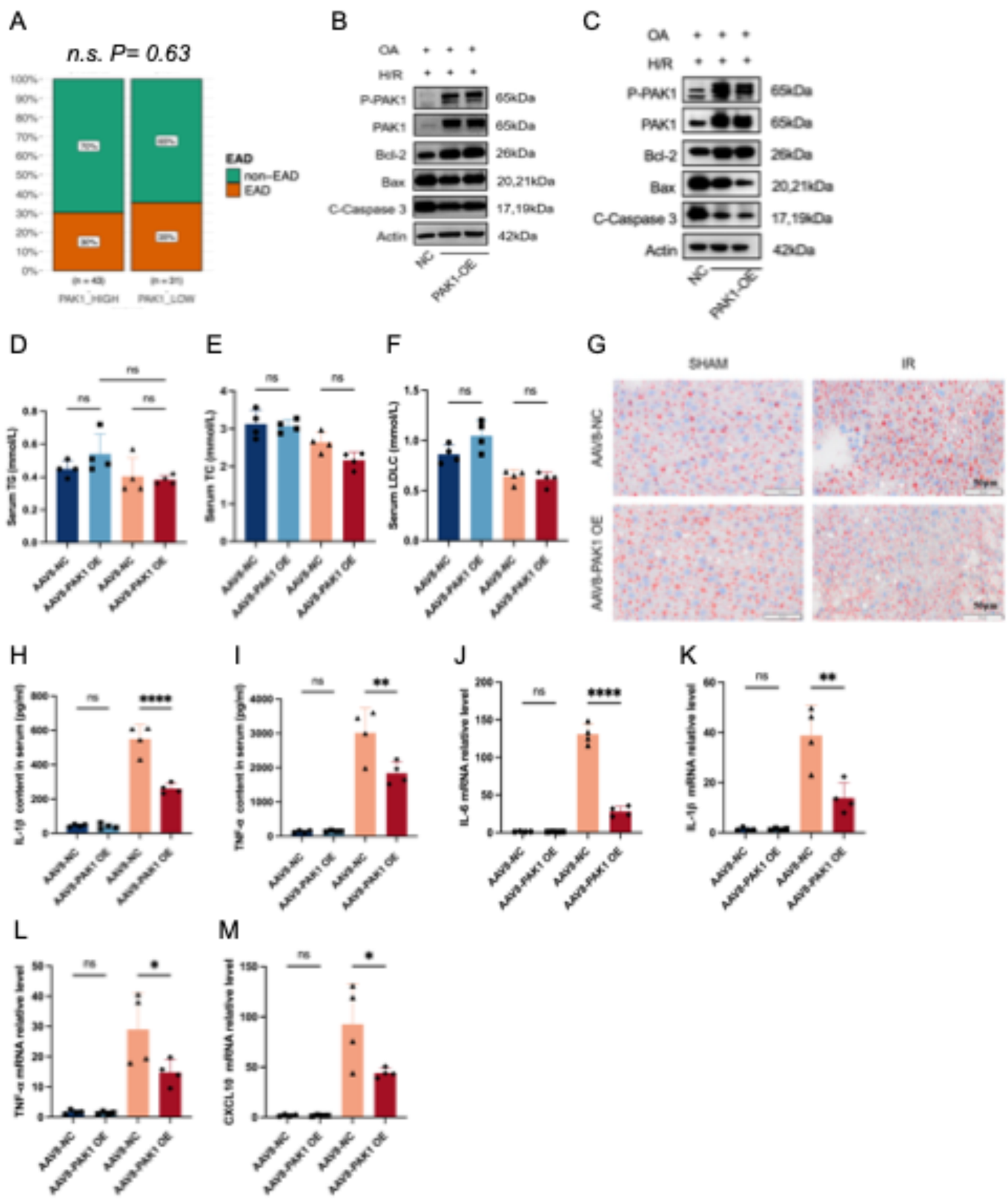

Figure S1 Clinical EAD analysis and complementary assessment of PAK1 overexpression in steatotic hepatic ischemia-reperfusion.

(A) Incidence of early allograft dysfunction (EAD) in PAK1-high (n = 43 grafts) and PAK1-low (n = 31 grafts) groups. (B, C) Additional representative immunoblots of P-PAK1, PAK1, Bcl-2, Bax, and cleaved caspase-3 in OA-treated AML12-NC and AML12-PAK1-OE cells after H/R; actin was the loading control. (D-F) Serum triglyceride, total cholesterol, and low-density lipoprotein cholesterol in AAV8-NC and

AAV8-PAK1-OE mice after sham surgery or IR (n = 4 mice per group). (G) Representative Oil Red O staining after sham surgery or IR. Scale bar, 50 µm. (H, I) Serum IL-1β and TNF-α after sham surgery or IR (n = 4 mice per group). (J-M) Hepatic IL-6, IL-1β, TNF-α, and CXCL10 mRNA after sham surgery or IR (n = 4 mice per group). Three independent cell experiments for Figure S1 B, C. One in vivo experiment using biologically independent mice. Data are mean ± SD. Fisher's exact test was used for A, and one-way ANOVA for D–F and H–M. ns, not significant; *P < 0.05; **P < 0.01; ****P < 0.0001. EAD, early allograft dysfunction; OA, oleic acid; H/R, hypoxia/reoxygenation; IR, ischemia-reperfusion; TG, triglyceride; TC, total cholesterol; LDL-C, low-density lipoprotein cholesterol.

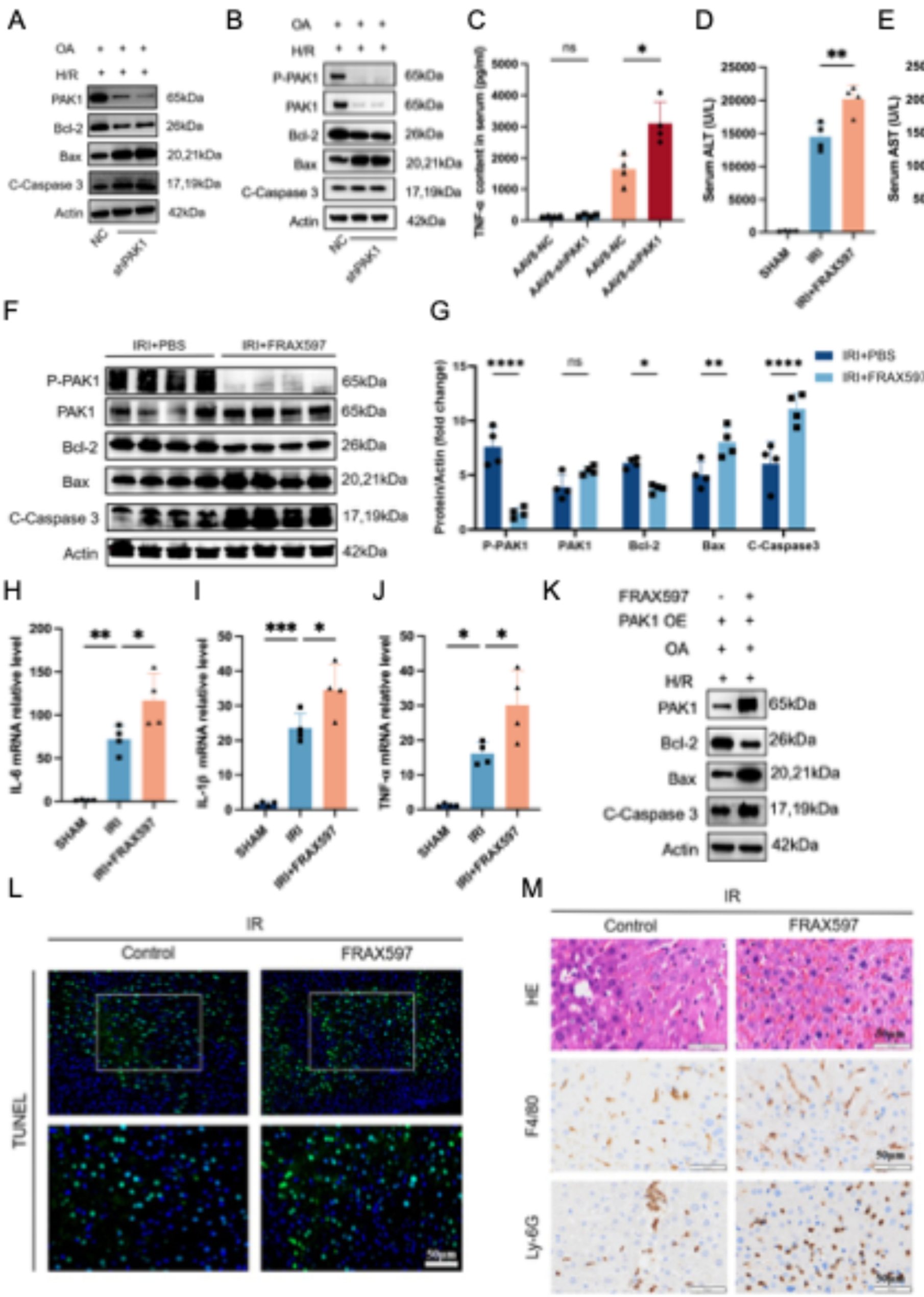

Figure S2 Complementary validation of PAK1 loss of function and pharmacological PAK inhibition during hepatic ischemia-reperfusion.

(A, B) Additional representative immunoblots of PAK1, P-PAK1, Bcl-2, Bax, and cleaved caspase-3 in OA-treated AML12-NC and AML12-shPAK1 cells after H/R; actin was the loading control. (C) Serum TNF-α in AAV8-NC and AAV8-shPAK1 mice after sham surgery or IR ($n = 4$ mice per group). (D, E) Serum ALT and AST in sham, IR, and IR+FRAX597 groups ($n = 4$ mice per group). (F, G) Representative liver immunoblots and quantification of P-PAK1, PAK1, Bcl-2, Bax, and cleaved caspase-3 after IR with vehicle or FRAX597 ($n = 4$ mice per group); actin was the loading control. (H-J) Hepatic IL-6, IL-1β, and TNF-α mRNA in sham, IR, and IR+FRAX597 groups ($n = 4$ mice per group). (K) Representative immunoblots of PAK1, Bcl-2, Bax, and cleaved caspase-3 in PAK1-OE AML12 cells after H/R with or without FRAX597. (L) Representative TUNEL staining after IR with vehicle or FRAX597. Scale bar, 50 μm. (M) Representative H&E, F4/80, and Ly6G staining after IR with vehicle or FRAX597. Scale bar, 50 μm. Three independent cell experiments for Figure S2 A, B. One in vivo experiment using biologically independent mice. Data are mean ± SD. One-way ANOVA was used for C–E and H–J, and two-way ANOVA for G. ns, not significant; $*P < 0.05$; $**P < 0.01$; $***P < 0.001$; $****P < 0.0001$. FRAX597, PAK inhibitor; IR, ischemia-reperfusion; ALT, alanine aminotransferase; AST, aspartate aminotransferase.

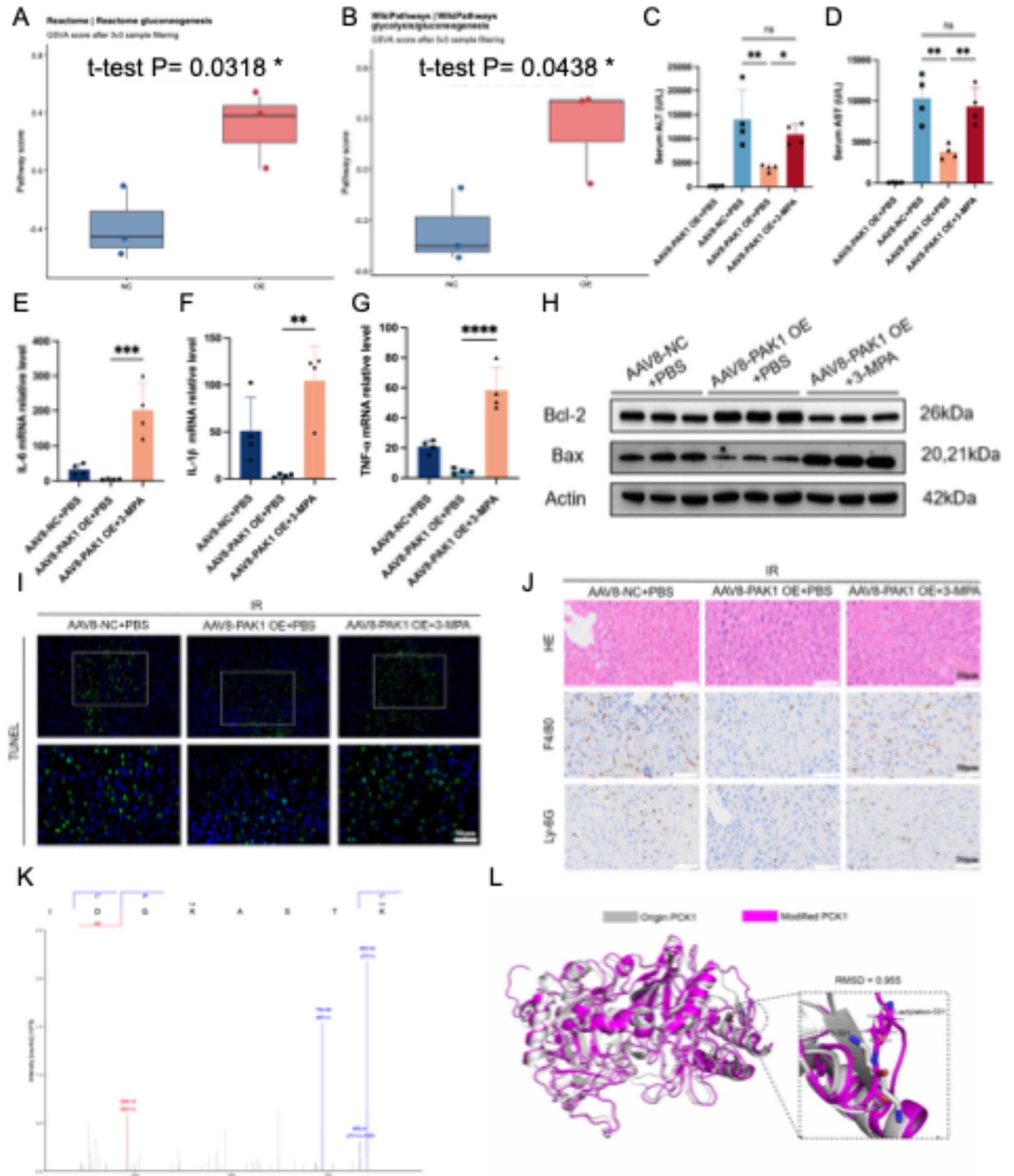

Figure S3 Pharmacological PCK1 inhibition attenuates the protective effects of hepatic PAK1 overexpression during steatotic hepatic ischemia-reperfusion.

(A, B) GSVA pathway scores for Reactome gluconeogenesis and WikiPathways glycolysis/gluconeogenesis in OA-treated control and PAK1-OE AML12 cells after H/R (n = 3 samples per group). (C, D) Serum ALT and AST levels in the four indicated mouse groups (n = 4 mice per group). (E-G) Hepatic IL-6, IL-1β, and TNF-α mRNA levels in AAV8-NC+PBS, AAV8-PAK1-OE+PBS, and AAV8-PAK1-OE+3-MPA mice after IR (n = 4 mice per group). (H) Representative liver immunoblots of Bcl-2 and Bax in the same groups (n = 3 mice per group); actin was the loading control. (I) Representative TUNEL staining of liver sections from the indicated groups after IR, shown as overview images and enlargements of the boxed regions (n = 4 mice per group). Scale bar, 50 μm. (J) Representative H&E, F4/80, and Ly6G staining of liver

sections from the indicated groups after IR (n = 4 mice per group). Scale bar, 50 μm. (K) Representative LC-MS/MS spectrum of a PCK1 peptide carrying lactylation at K547 and K551. (L) Structural superposition of native and K551-lactylated PCK1, with the modified region enlarged (RMSD = 0.955). One RNA-seq/GSVA experiment with three samples per group. Data in C-G are mean ± SD, with individual data points shown. Unpaired two-sided Student's t tests were used for A and B ($P = 0.0318$ and $P = 0.0438$, respectively), and one-way ANOVA was used for C-G. ns, not significant; *$P < 0.05$; **$P < 0.01$; ***$P < 0.001$; ****$P < 0.0001$. GSVA, gene set variation analysis; OA, oleic acid; H/R, hypoxia/reoxygenation; IR, ischemia-reperfusion; AAV8, adeno-associated virus 8; NC, negative control; OE, overexpression; PBS, phosphate-buffered saline; 3-MPA, 3-mercaptopicolinic acid; ALT, alanine aminotransferase; AST, aspartate aminotransferase; TUNEL, terminal deoxynucleotidyl transferase dUTP nick-end labeling; H&E, hematoxylin and eosin; PCK1, phosphoenolpyruvate carboxykinase 1; LC-MS/MS, liquid chromatography-tandem mass spectrometry; RMSD, root-mean-square deviation.

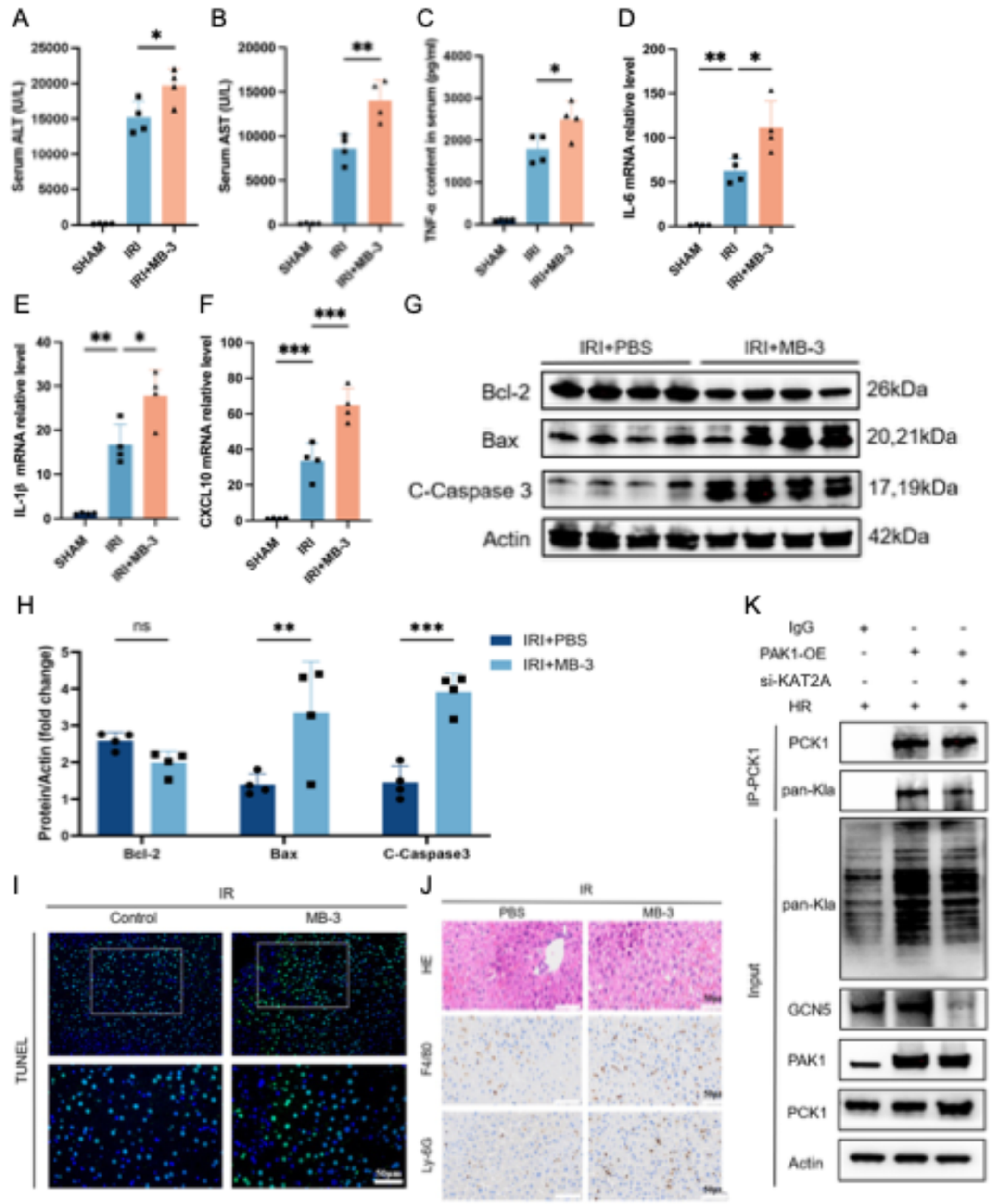

Figure S4 KAT2A inhibition exacerbates hepatic ischemia-reperfusion injury and reduces PAK1-associated PCK1 lactylation.

(A-C) Serum ALT, AST, and TNF-α in sham, IR, and IR+MB-3 groups (n = 4 mice per group). (D-F) Hepatic IL-6, IL-1β, and CXCL10 mRNA in the same groups (n = 4 mice per group). (G, H) Representative liver immunoblots and quantification of Bcl-2, Bax, and cleaved caspase-3 after IR with vehicle or MB-3 (n = 4 mice per group); actin was the loading control. (I) Representative TUNEL staining after IR with vehicle or MB-3. Scale bar, 50 μm. (J) Representative H&E, F4/80, and Ly6G staining after IR with vehicle or MB-3. Scale bar, 50 μm. (K) PCK1 immunoprecipitation followed by pan-Kla immunoblotting in PAK1-OE AML12 cells after H/R with or without KAT2A knockdown; IgG and input controls are shown. One in vivo experiment using

biologically independent mice. Data are mean ± SD. One-way ANOVA was used for A-F, and two-way ANOVA for H. ns, not significant; *P < 0.05; **P < 0.01; ***P < 0.001. MB-3, KAT2A inhibitor; KAT2A (also known as GCN5), lysine acetyltransferase 2A; PCK1, phosphoenolpyruvate carboxykinase 1; pan-Kla, pan-lysine lactylation.

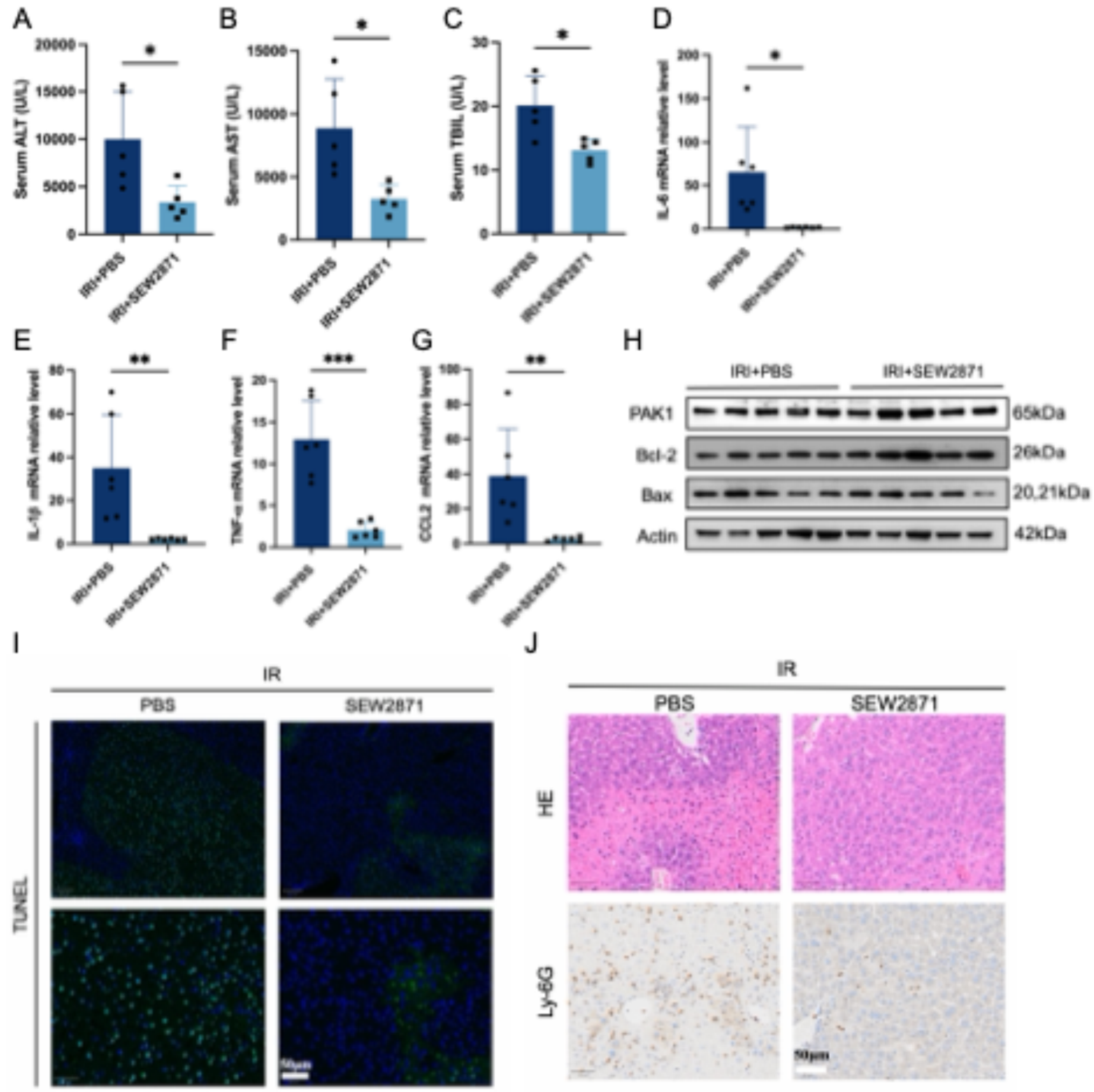

Figure S5 The S1PR1-selective agonist SEW2871 attenuates steatotic hepatic ischemia-reperfusion injury.

(A-C) Serum ALT, AST, and total bilirubin after IR with vehicle or SEW2871 (n = 5 mice per group). (D) Hepatic IL-6 mRNA after IR with vehicle or SEW2871 (n = 5 mice per group). (E-G) Hepatic IL-1β, TNF-α, and CCL2 mRNA after IR with vehicle or SEW2871 (n = 6 mice per group). (H) Representative liver immunoblots of PAK1, Bcl-2, and Bax after IR with vehicle or SEW2871 (n = 5 mice per group); actin was the loading control. (I) Representative TUNEL staining after IR with vehicle or SEW2871. Scale bars as indicated. (J) Representative H&E and Ly6G staining after IR with vehicle or SEW2871. Scale bar, 50 μm. One in vivo experiment using the indicated numbers of biologically independent mice. Data are mean ± SD. Unpaired two-sided Student's t

tests were used for A-G. *P < 0.05; **P < 0.01; ***P < 0.001. S1PR1, sphingosine-1-phosphate receptor 1; ALT, alanine aminotransferase; AST, aspartate aminotransferase; TBIL, total bilirubin; IR, ischemia-reperfusion.

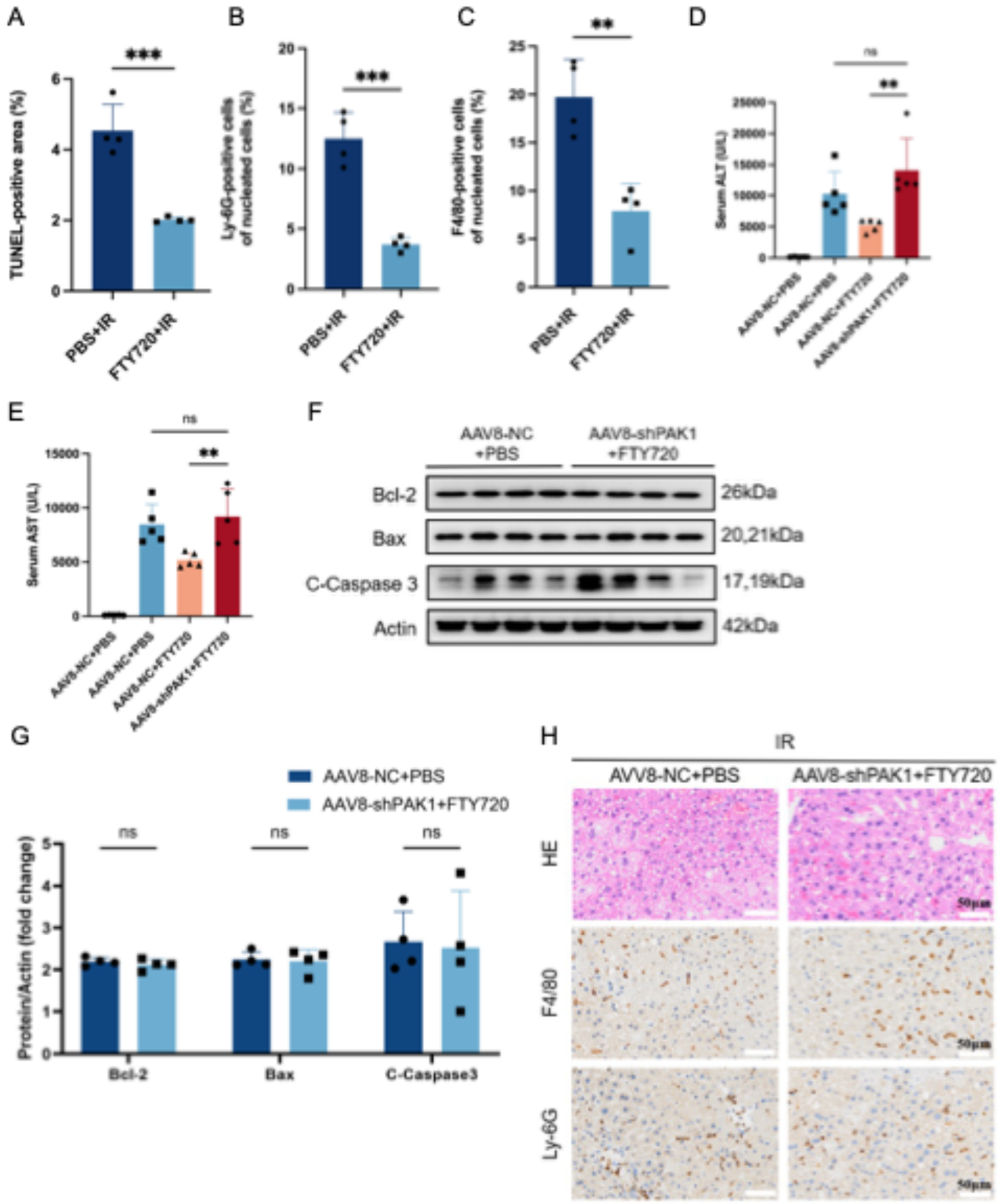

Figure S6 Hepatic PAK1 knockdown weakens the protective effects of FTY720 during steatotic hepatic ischemia-reperfusion.

(A-C) Quantification of TUNEL-positive area, Ly6G-positive cells, and F4/80-positive cells after IR with vehicle or FTY720 (n = 4 mice per group). (D, E) Serum ALT and AST in sham AAV8-NC+vehicle mice and in IR mice receiving AAV8-NC+vehicle, AAV8-NC+FTY720, or AAV8-shPAK1+FTY720 (n = 5 mice per group). (F, G) Representative liver immunoblots and quantification of Bcl-2, Bax, and cleaved

caspase-3 in IR AAV8-NC+vehicle and AAV8-shPAK1+FTY720 mice (n = 4 mice per group); actin was the loading control. (H) Representative H&E, F4/80, and Ly6G staining in the same groups. Scale bar, 50 µm. One in vivo experiment using the indicated numbers of biologically independent mice. Data are mean ± SD. Unpaired two-sided Student's t tests were used for A-C, one-way ANOVA for D and E, and two-way ANOVA for G. ns, not significant; **P < 0.01; ***P < 0.001. FTY720, fingolimod; AAV8, adeno-associated virus 8; shPAK1, short hairpin RNA targeting PAK1; ALT, alanine aminotransferase; AST, aspartate aminotransferase.

Supplementary Tables

Table S1. Plasmids and expression vectors used in this study

**Plasmids and Expression Vectors**	**Cat No.**	**Manufacture**
pLV3-CMV-PAK1(mouse)-3×FLAG-CopGFP-Puro	P85525	Miaoling Bio
pLV3-CMV-PAK1(human)-3×FLAG-CopGFP-Puro	P47371	Miaoling Bio
pLV3-CMV-ACSS2(human)-3×FLAG-EF1a-mCherry-Neo	P89668	Miaoling Bio
pCMV-ACSS2(human)-3×HA-Neo	P50020	Miaoling Bio
pCMV-KAT2A(human)-3×HA-WPRE-Neo	P92970	Miaoling Bio
pLV3-CMV-KAT2A(human)-3×FLAG-CopGFP-Puro	P81889	Miaoling Bio
pLV3-CMV-PCK1(human)-3×HA-Puro	P87757	Miaoling Bio
pLV3-CMV-PCK1(mouse)-3×FLAG-CopGFP-Puro	P57261	Miaoling Bio
pLV3-CMV-ACSS2 S267A(human)-3×FLAG-Neo	-	Miaoling Bio
pLV3-CMV-ACSS2 S659A(human)-3×FLAG-Neo	-	Miaoling Bio

Table S2. Primers for real-time qPCR detection

**Gene**		**Sequence5'---3'**
Mouse IL-6	**F**	CTGCAAGAGACTTCCATCCAG
	**R**	AGTGGTATAGACAGGTCTGTTGG
Mouse IL-1β	**F**	TTCAGGCAGGCAGTATCACTC
	**R**	GAAGGTCCACGGGAAAGACAC
Mouse TNF-α	**F**	CAGGCGGTGCCTATGTCTC
	**R**	CGATCACCCCGAAGTTCAGTAG
Mouse β-actin	**F**	GTGACGTTGACATCCGTAAAGA
	**R**	GCCGGACTCATCGTACTCC
Mouse CXCL10	**F**	AATGAGGGCCATAGGGAAGC

	**R**	AGCCATCCACTGGGTAAAGG

Table S3. Antibodies for immunoblot analyses

**Antibody**	**Cat No.**	**Manufacture**
**Cleaved caspase 3**	9661S	CST
**Bax**	2772S	CST
**Bcl2**	ab182858	Abcam
**PAK1**	21401-1-AP	Proteintech
**P-PAK1**	2601S	CST
**β-actin**	66009-1-Ig	Proteintech
**Flag**	66008-4-Ig	Proteintech
**HA**	51064-2-AP	Proteintech
**ACSS2**	16087-1-AP	Proteintech
**Pan Phospho-Serine/Threonine**	T91067	Abmart
**Anti-L-Lactyl Lysine Rabbit mAb**	1401RM	PTM
